# Supplementary material for: Work stress, work motivation and their effects on job satisfaction in community health workers: a cross-sectional survey in China
Source: BMJ Open. 2014 Jun 5;4(6):e004897. doi: 10.1136/bmjopen-2014-004897 (PMC4054641; doi:10.1136/bmjopen-2014-004897)
Supplement: Author's manuscript [file bmjopen-2014-004897.draft_revisions.pdf]

Work stress, work motivation and their effects on job satisfaction for community health workers in China

Journal:	*BMJ Open*
Manuscript ID:	bmjopen-2014-004897
Article Type:	Research
Date Submitted by the Author:	21-Jan-2014
Complete List of Authors:	Li, Li; School of Public Health, Harbin Medical University, Department of Health Management<br>Zhou, Hao; Center for Disease Control and Prevention, Department of Emergency<br>Hu, Hongyan; The Second Affiliated Hospital, Harbin Medical University, Research Service Office<br>He, Changzhi; School of Public Health, Harbin Medical University, Department of Health Management<br>Fan, Lihua; School of Public Health, Harbin Medical University, Department of Health Management<br>Liu, Xinyan; School of Public Health, Harbin Medical University, Department of Health Management<br>Zhang, Zhong; School of Public Health, Harbin Medical University, Department of Health Management<br>Li, Heng; School of Public Health, Harbin Medical University, Department of Health Management<br>Sun, Tao; School of Public Health, Harbin Medical University, Department of Health Management
**Primary Subject Heading**:	Health services research
Secondary Subject Heading:	Public health
Keywords:	Human resource management < HEALTH SERVICES ADMINISTRATION & MANAGEMENT, Organisational development < HEALTH SERVICES ADMINISTRATION & MANAGEMENT, Organisation of health services < HEALTH SERVICES ADMINISTRATION & MANAGEMENT

SCHOLARONE™
Manuscripts

Work stress, work motivation and their effects on job satisfaction for community health workers in China

Li Li*[1], Hao Zhou[2], Hongyan Hu[3], Changzhi He[1], Lihua Fan[1], Xinyan Liu[1], Zhong Zhang, Heng Li[1] ,Tao Sun[1]

[1] Department of Health Management, School of Public Health, Harbin Medical University, Harbin, China

[2] Department of Emergency, Center for Disease Control and Prevention, Harbin, China

[3] Research Service Office, The Second Affiliated Hospital, Harbin Medical University, Harbin, China

Correspondence to Li Li: lilihmu@gmail.com

Abstract

Objective: It has been well documented that both work stress and work motivation are key determinants of job satisfaction. The aim of this study is to examine the level of work stress and work motivation and their contribution to job satisfaction among community health workers in Heilongjiang Province, China.

Methods: A cross-sectional survey of 930 community health workers from six cities in Heilongjiang province, China, was conducted from

For peer review only

October01, 2012 to December 31, 2012. Multi-stage sampling procedures were used to measure socio-economic and demographic status, work stress, work motivation and job satisfaction. Logistic regression analysis was performed to assess key determinants of job satisfaction.

Results: There were significant differences in some subscales of work stress and work motivation by some of socio-economic characteristics. Dissatisfied respondents had significant higher levels in overall perception and five subscales of work stress than satisfied workers. However, satisfied respondents had higher levels in overall perception and five subscales of work motivation than dissatisfied respondents, with the exception of finance motivation. The main determinants of job satisfaction were occupation; age; title; income; the career development and wages & benefits subscales of work stress; and the recognition, responsibility and finance subscales of work motivation.

Conclusion: The finding of this study suggested that there is considerable room for improvement in job satisfaction among community health workers of Heilongjiang Province in China. Health care managers should take both work stress and work motivation into consideration, since two subscales of work stress and one subscale of work motivation negatively influenced job satisfaction and two subscales of work motivation positively influence job satisfaction.

Article summary

For peer review only

Article focus

▪What is the mean value of overall perception and subscales of work stress and work motivation in respect to the level of job satisfaction?

▪What is facet score of work stress and work motivation by socio-economic and demographic status?

▪How can work stress and work motivation influence job satisfaction among community health workers?

Key message

There is considerable room for improvement in job satisfaction among community health workers and health care managers should take both work stress and work motivation into consideration.

Strength and limitations of this study

This study is one of the first of its kind to examine the combined effects of work stress and work motivation on job satisfaction among urban community workers in China since the implementation of new health system reform. The instrument used in this study was not an international commonly scale and the survey was conduct by self-administrated method.

Introduction

In 2009，the Chinese central government promulgated a new health system reform plan and called for the development of community health service. As the foundation of the three-tier health system in China,

For peer review only

CHCs played a very important role in improving access to health care service, enhancing equity and reducing hospitalization and costs. [1-2] From 2009 to 2012， the number of community health institutions increased 6254 and the number of visits in them increased 193.949 million person-times. So, community health centers and workers thereof, are very important in the process of health system reform.

Heilongjiang Province is located in Northeast China with population of about 38.1 million. There are 776 urban community health institutions with 13100 health workers as of December 31, 2012.[3] However, limited resources, shortage of skilled health workers constituted a very important bottleneck to service and many of community health workers experienced work related stress and had low motivation.[4-5] Lots of research has shown that work stress and work motivation can greatly affect the workers' job satisfaction and in turn the quality and delivery of health care. While, few studies have specifically evaluated the level of work stress and motivation and their effects on job satisfaction among Chinese community health workers after the implementation of the new health system reform policy.

Work stress can be defined as the harmful physical and emotional responses that occur when job requirements do not match the worker's capabilities, resources, and needs of the workers and Cooper believed that stress resulted from a misfit between individuals and their environment.

For peer review only

[6-7] A survey conducted by international survey research of Chicago reported that forty percent of these peoples said they had too much pressure at work.[8] Kazufumi, et al identified major work stress factors in an organization.[9-11] Lots of research has been conducted on the relationship between work stress and job satisfaction and found kinds of work related stress lead to job dissatisfaction.[10,12-15]

Work motivation can be defined as an individual's degree of willingness to exert and maintain an effort towards attaining organizational goals and Nahavandi and Malekzadeh believed that motivation was a driver of stable mind, aspiration or interest within the individual that can translate into action.[16-18] It can be inferred from these definitions that to motivate workers is to stimulate them or cause them to desire to do something. Patrick and Wilbroad developed a tool to measure health worker motivation and revealed the major determinants of higher motivation.[19-20] Tribolet explored the relationship between intrinsic and extrinsic motivation.[21] Pool found significant positive association between work motivation and job satisfaction, whereas Stringer revealed that intrinsic motivation was positively associated with job satisfaction and extrinsic motivation was negatively associated with job satisfaction.[22-23]

In China, Ge (2011) analyzed the relationship between work stress and job satisfaction among Chinese community health workers and

For peer review only

identified key predictors of job satisfaction.[24] Chen (2012) investigated the relationship between work motivation, work stress and job satisfaction toward cross-strait employees in Taiwan and mainland China.[25]

This study focused on the major factors of work stress and motivation demonstrated in research findings and provided an overview from community health workers' perspective of work stress and motivation factors.[11, 26 -28] The purpose of this study was to assess the determinants of job satisfaction among community health workers in one Chinese province. A cross-sectional survey was conducted to measure the level work stress, work motivation and job satisfaction. The key determinants of job satisfaction for community health workers were assessed with special attention devoted to work stress and work motivation.

Design and methods

Samples

A cross-sectional survey was conducted aiming at community health workers during March 1st and October 31, 2013 in Heilongjiang Province, China. A multi-stage, stratified sampling design was employed to ensure study data were provincially representative. First, 6 cities (Harbin, Qiqihar, Suihua, Jiamusi, Qitaihe, Heihe) were selected based on GDP figures in three levels. Second, 15 community health centers were

For peer review only

randomly selected from each city. The research team visited the selected community health centers and invited all general practitioners, public health physician, nurses and other health technical staff to participate in the study with the exception of those who were sick and absent. The survey questionnaires were completed by respondents themselves in order to ensure confidentiality. The research staff stayed in a room of the community health center for a whole day and was available to answer any respondents' questions. Therefore, respondents can choose their appreciate time to complete the questionnaire (such as, when they were not busy or their office was quiet). Finally, 980 community health workers participated in the survey and the self-administrative questionnaire was completed by all study subjects, yielding a response rate of 100%. In total, there were 930 respondents but of 50 (5.1%) were incomplete. This study was approved by Medical Ethic Committee of Harbin Medical University.

Assessment tools

The study instrument was a self-administered questionnaire and was composed of 4 sections. Section 1 focused on the socio-economic and demographic status of respondents.

Section 2 was used to assess the value of work stress with a 30-item instrument developed through qualitative intensive interviews with health

For peer review only

care managers and community health workers, review of literatures and an initial pilot study.[11,26] These items were divided into five subscales by factor analysis, which didn't be discussed here. These five subscales of work stress were named as work task & role stress, career development stress, wages & benefits stress, working relationship stress, and organizational structure & climate stress respectively. Respondents were asked to rate their perception of work stress on each item based on a 5-point Likert scale, very less stressful (1), less stressful (2), average (3), stressful (4) and very stressful (5). The Cronbach's alpha value for this study was 0.87.

Section 3 was used to assess work motivation. The four subscales of work motivation, as captured in previous research and identified by factor analysis (factor analysis didn't be discussed here), were career development motivation, recognition motivation, responsibility motivation and finance motivation.[27-29] In this study, we referred to career development motivation and finance motivation as extrinsic motivation, and recognition motivation and responsibility motivation as intrinsic motivation.[23,28] Respondents were asked to rate their motivation intensity on each item based on a 5-point Likert scale, very less strong (1), less strong (2), average (3), strong (4) and very strong (5). The Cronbach's alpha value for this study was 0.75.

Section 4 was used to assess job satisfaction. In this study, a

For peer review only

single-item measure was adopted in measuring overall job satisfaction.[30] The respondents were asked to indicate their level of job satisfaction on a 4-point Likert scale, strongly dissatisfied (1), dissatisfied (2), satisfied (3) and dissatisfied (4).

Data analysis

Survey results were analyzed using SPSS 17.0. Descriptive analyses included frequencies and percentages for categorical variables, means and standard deviations (SDs) for continuous variables. Mean differences were examined using t-test and ANOVA for relevant subgroups. And, logistic regression was used to measure key determinants of job satisfaction.

Results

Socio-economic and demographic status of respondents

Socio-economic and demographic status of the sample were shown in Table 1. A majority of the participants were female (74.6%). General practitioners accounted for 36% of community health workers surveyed, followed by nurses (28.8%), public health physician (19.1%). In this survey, only 18.6% of them had senior professional titles and less than half (40.2%) of them had bachelor degree or higher. Only 19.6% of them had monthly incomes of more than 3,000 RMB (where $1.00US=6.23RMB in 2012). Nearly ninety percent of respondents worked more than 40 hours per week.

For peer review only

Facet scores of work stress and work motivation by socio-economic and demographic status

Results of variance analysis and further multiple comparison t-test were showed in Table 1. It indicated that there was significant difference in all of the five subscales of work stress by occupation ($p<0.01$) and sex ($p<0.05$), with general practitioners and male having higher levels of work stress. The wages & benefits subscale of work stress showed significant difference by educational background ($p<0.05$) and income ($p<0.05$). Respondents with middle professional title had significant higher level of stress in work task & role subscale ($p< 0.01$) and in relationship subscribe ($p<0.05$). Those who were aged 35-44 and 45-54 years had significant higher level of stress in task & role subscale ($p< 0.01$).

There was no significant difference in all of the four subscales of work motivation by educational background, professional title and income. The male had significant higher level recognition and financial motivation ($p<0.05$). Younger workers (<25) had significantly higher level of recognition motivation ($p<0.05$) and responsibility motivation ($p<0.05$). A higher level of recognition motivation was expressed by general practitioners ($p<0.05$).

Level of work stress and work motivation in respect to the level of job satisfaction

For peer review only - http://bmjopen.bmj.com/site/about/guidelines.xhtml

Table 2 revealed mean score of overall perception of work stress was 3.11，which was only above the mid-point of 3. Wages & benefits (3.60) subscale of work stress ranked in the highest position, followed by work task & role (3.31), career development (2.96), organizational structure & climate (2.90) and relationship (2.75) subscales of work stress. (F=154.9，$p<0.001$). Statistically significant differences were noted in overall perception and the five subscales of work stress between the satisfied and dissatisfied groups of respondents, with those who were dissatisfied having higher levels of work stress ($p<0.001$).

Career development motivation was rated the highest level, followed by financial, recognition and responsibility motivation (F=202.6，$p<0.001$). Levels of overall perception of work motivation and all subscales with the exception of financial motivation were significantly different between the satisfied and dissatisfied groups of respondents, and the satisfied workers had higher levels of work motivation ($p<0.01$).

Determinants of job satisfaction

In our study, 61.3% of respondents were satisfied with job. Table 3 presented results from logistic regression model that examined key determinants of job satisfaction with the special attention devoted to work stress and work motivation.

Results demonstrated that only a few demographic characteristics were determinants of job satisfaction. And we found that when career

For peer review only

development and wages & benefits subscales of work stress increased one grade, job satisfaction decreased 32% [odds ratio (OR) =0.68, p<0.05] and 37% (OR= 0.63, p<0.01) respectively. When finance motivation increased one grade, job satisfaction would decrease 28% (OR=0.72, p<0.01). Whereas, when recognition motivation and responsibility motivation increased one grade, job satisfaction would increase 1.86 timeshare (OR=2.86, p<0.01) and 0.36 times (OR=1.36, p<0.05) respectively. Compared with nurses, general practitioners (OR=0.56, p<0.01) and public health physician (OR=0.42, p<0.05) had lower job satisfaction, while other technical staff (OR=1.89) had higher level of job satisfaction. Workers with no title (OR=7.02, p<0.05) were more satisfied than workers with senior title.

Discussion

This study was one of the first of its kind to examine the level of work stress and work motivation and their combined effects on job satisfaction among urban community workers in China since the implementation of new health system reform. These findings have significant implications for managers in their efforts to improve workers' job satisfaction.

First, managers should pay more attention to reduce workers work stress. Many of previous research have focused on the relationship between overall work stress and job satisfaction, while this study examined the level of five subscales of work stress and their effects on

For peer review only

job satisfaction.[31-32] The results indicated that mean scores of the five subscales of work stress in dissatisfied respondents were significant higher than those in satisfied respondents. And the career development and wages & benefits subscales of work stress were negatively related with job satisfaction. The findings were consisted with previous studies that workers were likely to report low job satisfaction if they did not receive promotion and advancement opportunities and did not get adequate salary.[33-34] But this was different from some other studies. McGown found interpersonal relationships were major stressors reported by workers, and Lee and Callaghan found work overload was the commonest faced by most nurses.[35-37] It should be concerned that in this study these two negative determinants of job satisfaction were ranked first and third highest level among five subscales respectively.

Second, managers should take measures to inspire workers intrinsic motivation. In this study, we referred to career development and finance motivation as extrinsic motivation, while recognition and responsibility motivation as intrinsic motivation based on literatures.[23,38] We found that the recognition and responsibility subscales of work motivation were positive determinants of job satisfaction and finance motivation was negative determinant. This was consistent with Becchetti's argument that when workers don't work for financial incentive, they may seek satisfaction irrespectively of the level of pay, even if the financial

For peer review only

incentive is kept to a minimum, workers may be satisfied with their job.[38] The "crowding-in" effect also formulated that the intrinsic motivation increases job satisfaction, whereas extrinsic motivation decreases job satisfaction (Frey, 1997).[39] It should be noted that in this study the level of extrinsic motivation was higher than intrinsic motivation.[40] This finding was consistent with Dermer's study, whereas contrary to Tribolet's study.[41, 21]

Several reasons might have contributed to these above findings. In Heilongjiang Province the average income of health service persons in urban units was 52,564 RMB (where $1.00US=6.23RMB in 2012) as of 2012. But in this study 80.4% of respondents' yearly income was less than 36,000 RMB. The poor salary increased their wages & benefits stress and finance motivation.[42] In the meanwhile, it was difficult for community health workers to get title promotion, for there were limit promotion quotas for CHCs every year in Heilongjiang Province and our study found only 18.6% of respondents had senior professional title.

As some subscales of work stress and work motivation can positively or negatively influence job satisfaction, we examined the different level of work stress and work motivation by demographic characteristics and found that managers should pay more attention to three kinds of workers. The first group workers were those aged 35-44 and 45- 54 years, who had higher level of stress in work task & role subscale and lower level of

For peer review only

intrinsic motivation. Similar results have been reported in Uganda, where the middle age groups was significantly more stressed than the youngest age group.[15] This could be related to workload, difficulties and complexity of the duties, which was usually more for 35-54 years age workers as they were the backbone of community health service. The second group was males. In our study, mean scores of all work stress subscales for males tended to be higher than that for females. Consistent with Malik's study, males had higher level in finance motivation.[43] But a British study of general practitioners indicated no differences in stress rates between males and females and David found female has more stress in financial rewards and role ambiguity.[44-45] The third group workers were general practitioners, who experienced highest stress in all of the five subscales and had highest career development motivation. In community health centers, general practitioners faced more difficult and complicated tasks and kinds of medical risks than others, and they had lower income and less promotion opportunities than those physicians in general hospitals.

Limitations of this study

The findings in this study need to be viewed in light of three key limitations. First, the instrument for assess the work stress and work motivation was developed from earlier study and discussed with experts, while not an international commonly scale. Second, we used a

For peer review only

cross-sectional survey, which may limit our ability to identify causal relationships between work stress and motivation and job satisfaction. Third, the measurements were conducted by self-administrated method and respondents' cognition can be affected by emotions at that point in time. So the common method bias and self-administrated bias might affect the results.

Conclusion

It is important for health-care managers to improve job satisfaction of health workers in low-resource settings. In this study, we comprehensively examined the level of work stress and work motivation by demographic characteristics and in respect to the level of job satisfaction, and additionally, the key determinants of job satisfaction were assessed using logistic regression analysis. The results indicated that community health workers rated wages & benefits highest among five subscales of work stress and workers extrinsic motivation were higher than intrinsic motivation. The career development and wages & benefits subscales of work stress and finance motivation were significant negative determinants of job satisfaction, whereas the recognition and responsibility subscales of motivation were significant positive determinants.

The study findings suggested that there is considerable room for improvement in job satisfaction in community health workers of

For peer review only

Heilongjiang Province in China and health care managers should take both work stress and work motivation into consideration. First they should pay more attention to three kinds of workers as they had higher work stress and extrinsic motivation. Second they should take a variety of measures to reduce career development and wages & benefits stress, as they were negative determinants of job satisfaction. Third, it is important for managers to inspire workers intrinsic motivation as it can positively influence job satisfaction.

For peer review only

Table 1 Facets of work stress and work motivation by socio-economic and demographic status for respondents

			Work stress					Work motiva
	N	%	work task &role	career development	wages &benefits	relationship	organizational structure & climate	recognition
**Occupation**								
**General practitioner**	335	36.0	3.53	3.17	3.78	2.90	3.14	3.61
**Public health physician**	178	19.1	3.20	2.89	3.70	2.63	2.96	3.57
**Nurse**	267	28.8	3.24	2.95	3.54	2.76	2.78	3.53
**Other**	150	16.1	3.09	2.79	3.45	2.65	2.84	3.59
**F**			6.91**	4.97**	3.45**	3.05**	6.25**	0.66
**Sex**								
**Male**	236	25.4	3.44	3.10	3.77	2.88	3.12	3.71
**Female**	694	74.6	3.27	2.93	3.56	2.72	2.85	3.56
**F**			2.50*	2.27*	2.60*	2.51*	4.09*	2.36*
**Educational background**								
**High school or below**	110	11.8	3.18	2.90	3.36	2.81	2.72	3.57
**Junior college**	446	48.0	3.28	2.94	3.61	2.74	2.86	3.57
**College and above**	374	40.2	3.36	3.00	3.65	2.73	3.16	3.60
**F**			2.30	0.66	4.21*	0.45	4.02*	0.13
**Age in years**								
**<25**	78	8.4	3.08	2.81	3.45	2.60	2.77	3.80
**25-34**	258	27.7	3.21	2.94	3.63	2.72	2.91	3.65
**35-44**	329	35.4	3.36	2.98	3.55	2.78	2.88	3.52
**45-54**	234	25.2	3.43	3.02	3.69	2.79	2.94	3.53
**⩾55**	31	3.3	3.12	2.88	3.54	2.71	2.93	3.48

F			4.71**	1.01	1.36	1.12	0.83	2.89*
**Title**								
Senior title	42	4.5	3.12	3.11	3.38	2.69	2.73	3.37
Vice-senior title	131	14.1	3.32	2.92	3.65	2.63	2.93	3.46
Middle title	399	42.9	3.43	3.03	3.69	2.85	2.94	3.56
Primary title	299	32.2	3.20	2.93	3.54	2.72	2.87	3.62
No title	59	6.3	3.23	2.86	3.48	2.58	2.89	3.73
F			3.96**	1.07	1.71	3.04*	0.59	1.73
**Monthly income （RMB）**								
<2000	361	38.9	3.24	2.95	3.69	2.76	2.90	3.61
2000-2999	386	41.5	3.32	2.96	3.61	2.75	2.88	3.59
3000-3999	139	14.9	3.43	2.97	3.44	2.68	2.96	3.52
≥4000	44	4.7	3.39	3.03	3.21	2.93	2.78	3.44
F			2.11	0.99	3.14*	2.11	0.99	0.54
**Working hours (per week)**								
<40 小时	110	11.8	3.27	2.82	3.52	2.82	2.94	2.96
40-47 小时	509	54.7	3.26	2.73	3.59	2.73	2.95	2.87
48-55 小时	250	26.9	3.36	2.71	3.62	2.71	2.93	2.89
≥56 小时	61	6.6	3.52	2.93	3.75	2.93	3.36	3.13
F			0.06	0.20	0.48	0.20	0.01*	0.11

*p<0.05 **p<0.01

Table 2 Mean scores of the overall perception and subscales of work stress and work motivation in respect to the level of job satisfaction

	Total (n=930)	Level of job satisfaction		
	Mean±SD	Satisfied (n=570, 61.3%)	Dissatisfied (n=360, 38.7%)	P
**Work stress**				
Overall perception ※	3.11±0.68	2.95±0.68	3.37±0.60	P=0.000
work task & role¶	3.31±0.81	3.18±0.82	3.52±0.76	P=0.000
career development¶	2.96±0.87	2.79±0.85	3.22±0.83	P=0.000
Wages& benefits ¶	3.60±0.95	3.38±0.94	3.95±0.85	P=0.000
relationship¶	2.75±0.79	2.61±0.79	2.96±0.74	P=0.000
organizational structure& climate¶	2.90±0.79	2.74±0.79	3.15±0.71	P=0.000
**Work motivation**				
Overall perception◎	3.80±0.55	3.86±0.55	3.70±0.55	P=0.000
Career development†	4.13±0.57	4.24±0.51	3.95±0.62	P=0.000
Recognition†	3.58±0.77	3.66±0.77	3.45±0.77	P=0.000
Responsibility†	3.45±0.77	3.53±0.77	3.32±3.52	P=0.000
Finance†	4.06±0.79	4.02±0.79	4.12±0.80	P=0.295

For peer review only

※ Mean score of overall perception of work stress was calculated for each respondent by adding the value of each item of work stress and then divided by the numbers of all item.

◎Mean score of overall perception of work motivation was calculated for each respondent by adding the value of each item of work motivation and then divided by the numbers of the item.

¶Mean score of each subscale of work stress was calculated for each respondent by adding the value of each item belongs to the subscale of work stress and then divided by the numbers of the item.

†Mean score of each subscale of work motivation was calculated for each respondent by adding the value of each item belongs to the subscale of work motivation and then divided by the numbers of the item.

Table 3 The logistic regression analysis for job satisfaction*

		B	Odds Ratio	95% CI	P
**Occupation (Reference : nurse)**	General practitioner	-0.89	0.56	0.38-0.81	0.001
	Public health physician	-1.24	0.42	0.20-0.87	0.021
	Other technical staff	1.48	1.89	1.04-3.44	0.030
**Sex (Reference :male)**	Female	0.36	1.27	0.83-1.95	0.268
**Educational background (Reference :High school or below)**	Junior college	-0.26	0.76	0.43-1.34	0.342
	College and above	-0.29	0.75	0.41-1.40	0.373
**Age in years (Reference : <25)**	25-34	-0.50	0.60	0.30-1.21	0.151
	35-44	0.10	1.10	0.51-2.42	0.796
	45-54	0.03	1.04	0.45-2.35	0.926
	⩾55	2.14	8.53	1.86-39.01	0.006
**Title (Reference : senior title)**	Vice-senior title	0.65	1.86	0.476-7.29	0.371
	Middle title	0.99	2.57	0.67-9.78	0.165
	Primary title	1.23	3.84	0.96-15.39	0.057
	No title	1.94	7.02	1.53-32.12	0.012
**Monthly income in RMB (Reference : <2000)**	2000-2999	-0.68	0.50	0.26-0.98	0.051
	3000-3999	-0.01	0.99	0.64-1.52	0.973
	⩾4000	0.26	1.30	0.86-1.97	0.201
**Weekly hours worked (Reference :<40)**	40-47	-0.10	0.90	0.59-1.37	0.630
	48-55	0.06	1.07	0.67-1.70	0.773
	⩾56	0.18	1.20	0.62-2.33	0.582
**Work stress**	work task & role	-0.04	0.98	0.74-1.300	0.915
	career development	-0.36	0.68	0.49-0.94	0.020
	wages & benefits	-0.52	0.63	0.50-0.79	0.000
	relationship	-0.17	0.80	0.59-1.09	0.166
	Organizational structure & climate r	-0.03	0.97	0.71-1.33	0.881

For peer review only

Work motivation	Career development	0.17	1.13	0.85-1.505	0.399
	Recognition	1.03	2.86	2.02-4.04	0.000
	Responsibility	0.30	1.36	1.02-1.81	0.035
	Finance	-0.29	0.72	0.56-0.92	0.009

*Strongly satisfied and satisfied coded as 1 vs. strongly dissatisfied and dissatisfied coded as 0.

For peer review only

Acknowledgements We are thankful to all the community health workers who participated in the study. We are also grateful to Yin Li, Xingsan Li, Zhuang Wang and Hongjuan Wei, who have worked closely with the team to ensure the field survey is successfully implemented.

Completing interests None.

Contributors

LL was responsible for the study design, data analysis and the drafting and revising of the manuscript. HH and CH, who contributed equally as the first author to this article, were responsible for study design, data collection and data analysis. HZ and ZZ provided statistical expertise. XL, TS and HL performed data collection and technical support. LF provided administrative support. All authors read and approved the final manuscript.

Funding This study was funded by the National Science Foundation of China (NSFC), Contract No.71203050/G0308; and was supported by Young Seed Foundation of Public Health College of Harbin Medical

University. The opinions expressed herein are the authors' and do not necessarily reflect the views of NSFC and the survey was conducted independently by researchers from Harbin Medical University.

References

1. Starfield B, Shi LY, Macinko J.Contribution of primary care to health systems and health. Milbank Q 2005;83: 457–502.
2. Hung LM, Rane S, Tsai J, et al. Advancing primary care to promote equitable health: implications for China. Int J Equity Healt 2012;11:2.
3. Heilongjiang Provincial Bureau.Heilongjiang Health Statistical Yearbook 2012. Harbin, China: Heilongjiang Provincial Press 2013.
4. Zhou W, Dong YM, Lin XZ, et al. Community health service capacity in China: a survey in three municipalities. J Eval Clin Pract 2013;19:167–172.
5. Li L, Zhou H, Yao GF, et al. Analysis on working motivation of community health workers in Harbin, China. Medicine and Society 2013;7:40-43.
6. Park, Jungwee. Work stress and job performance. Per Lab Inco,2008; 20:7-19.

For peer review only

7. Cooper CL, Cartwright S. Healthy mind; healthy organization.-A proactive approach to occupational stress. Human Rel 1994;47(4):455.
8. Shellenbarger S. Are saner workloads the unexpected key to more productivity. Wall Str J 1999;10.
9. Kazufumi M, Kumiko S, Hiroki F, et al. Stressor scale for clinical research coordinators: development and psychometric testing. J Adv Nurs 2012; 2:1636-1645.
10. Kwok BC, Gina L, Yiu CK, et al. Work stress among six professional groups: the Singapore experience. Soc Sci Med 2000;50:1415-1432.
11. Cooper CL, Marshall J. Occupational sources of stress: a review of the literature relating to coronary heart disease and mental ill health. J Occu Psychol 1976;49:441-461.
12. Flanagan NA, Flanagan Timothy J. An analysis of the relationship between job satisfaction and job stress in correctional nurses. Res Nurs Health 2002;25(4):282–294.
13. Sveinsdottir H, Biering P, Ramel A. Occupational stress, job satisfaction, and working environment Icelandic nurses: a cross-sectional questionnaire survey. Int J Nurs Stud 2006; 43(7):875–889.
14. Zangaro GA, Soeken KL. A Meta-analysis of studies of nurses job

For peer review only

satisfaction. Res Nurs Health 2007;30, 445–458.

15. Nabiryephd RC, Brown KC, Pryor ER, et al. Occupational stress, job satisfaction and job performance among hospital nurses in Kampala, Uganda. J Nurs Manag 2011;19,760-768.

16. Franco LM, Bennett S, Kanfer R. Health sector reform and public sector health worker motivation: a conceptual framework. Soc Sci Med 2002;54:1255–1266.

17. Nahavandi, Afsaneh, Malekzadeh, et al. Organizational behavior: The person-organization fit, Prentice Hall 1999.

18. Kanfer R. Measuring health worker motivation in developing countries. Partnerships for Health Reform Project, Major Applied Research Working Paper 1999.

19. Patrick MM, Duane B, Lucy G, et al. Developing a tool to measure health worker motivation in district hospitals in Kenya. Hum Resour Health 2009; 7:40.

20. Wilbroad M, Helen A, Virginia B, et al. Measuring health workers' motivation in rural health facilities: baseline results from three study districts in Zambia. Hum Resour Health 2013;11,8.

21. Tribolet WC. The relationship between intrinsic and extrinsic motivation and organizational commitment: a study in a European environment [D]. Nova Southeastern Univ 2004.

22. Pool SW. The relationship of job satisfaction with substitutes of

For peer review only

leadership, leadership behavior, and work motivation. J Psychol 1997;131(3):271-83.

23. Stringer C, Jeni D, Theivananthampillai. Motivation, pay satisfaction, and job satisfaction of front-line employees. Qual Res Acco Man 2011; 8(2):161-179.
24. Ge C, Fu JL, Chang Y, et al. Factors associated with job satisfaction among Chinese community health workers: a cross-sectional study. BMC Public Health, 2011;11:884.
25. Chen TL, Huang MY, Su TH. Work motivation, work stress, and job satisfaction in between Taiwan and China-An empirical study. World Acad Sci, Eng Tech 2012; 68, 1446-1450.
26. Ajayi MP., Abimbola OH. Job satisfaction, organizational stress and employee performance: a study of NAPIMS. Ife Psychol 2013;21:75-82.
27. Wayne SJ., Liden RC. Effects of impression management on performance ratings: a longitudinal study. Acad Manage J 1995;38:232-260.
28. Gagne M, Deci EL. Self-determination theory and work motivation. J Organ Behav 2005;26:331-362.
29. Dieleman M, Cuong PV, Anh, LV, et al. Identifying factors for job motivation of rural health workers in North Vietnam. Hum Resour Health 2003;1:10.

For peer review only

30. Wanous JP, Reichers AE, Hudy MJ. Overall job satisfaction: how good are single-item measures? J Appl Psychol 1997;82:247–52.

31. Blau JR, Light, SC, Chamlin M. Individual and contextual effects on stress and job satisfaction: A study of prison staff. Work Occup 1986;13, 131-156.

32. Byrd TG, Cochran JK, Silverman IJ, et al. Behind bars: An assessment of the effects of job satisfaction, job-related stress, and anxiety on jail employees' inclinations to quit. J Cri Justice 2000; 23:69-89.

33. Lephalala RP. Factors influencing nurses job satisfaction in selected private hospitals in England. Curationis 2008;31(3): 60–69.

34. Carr KK, Kazanowski MK. Factors affecting job satisfaction of nurses who work in long-term care. J Adv Nurs 1994;19:878-883.

35. McGowan B. Self-reported stress and its effects on nurses. Nurs Stand 2001;15(42):33–38.

36. Lee I. Wang HH. Perceived occupational stress and related factors in public health nurses. J Nurs Res 2002;10(4):253–259.

37. Callaghan P, Tak-Ying SA, Wyatt PA. Factors related to stress and coping among Chinese nurses in Hong Kong. J Adv Nurs 2000;31(6), 1518 –1527.

38. Becchetti L, Castriota S, Tortia EC. Productivity, wages and intrinsic motivations. Sma Bus Eco 2012;41:379–399.

For peer review only

39. Frey BS. On the relationship between intrinsic and extrinsic work motivation. Int J Ind Organ 1997;15(4),427-439.

40. Taylor FW. Shop management. New York: Harper 1911.

41. Dermer, Jerry. The interrelationship of intrinsic and extrinsic motivation. Acad Manage J 1975;18(1):125-129.

42. National Bureau of Statistics. China Statistic Yearbook 2013. Beijing ,China: China Sta Press. 2013.

43. Malik AA, Yamamoto SS, Souares A, et al. Motivational determinants among physicians in Lahore Pakistan. BMC Health Serv Res 2010;10:201.

44. Rout U. Gender differences in stress, satisfaction and mental wellbeing among general practitioners in England. Psychol Health Med. 1999;4(4):345-354.

45. David PH, Srinika DJ. Gender differences in work stress among clinical social workers. J Soc Serv Res 2008;10:18.

For peer review only

STROBE Statement—Checklist of items that should be included in reports of ***cross-sectional studies***

	Item No	Recommendation	Reported on page #
**Title and abstract**	1	(*a*) Indicate the study's design with a commonly used term in the title or the abstract	2，7
		(*b*) Provide in the abstract an informative and balanced summary of what was done and what was found	2
**Introduction**			
Background/rationale	2	Explain the scientific background and rationale for the investigation being reported	4，,5
Objectives	3	State specific objectives, including any prespecified hypotheses	6
**Methods**			
Study design	4	Present key elements of study design early in the paper	7
Setting	5	Describe the setting, locations, and relevant dates, including periods of recruitment, exposure, follow-up, and data collection	7
Participants	6	(*a*) Give the eligibility criteria, and the sources and methods of selection of participants	7,8
Variables	7	Clearly define all outcomes, exposures, predictors, potential confounders, and effect modifiers. Give diagnostic criteria, if applicable	9-12
Data sources/ measurement	8*	For each variable of interest, give sources of data and details of methods of assessment (measurement). Describe comparability of assessment methods if there is more than one group	
Bias	9	Describe any efforts to address potential sources of bias	7
Study size	10	Explain how the study size was arrived at	7
Quantitative variables	11	Explain how quantitative variables were handled in the analyses. If applicable, describe which groupings were chosen and why	7-9
Statistical methods	12	(*a*) Describe all statistical methods, including those used to control for confounding	7-9
		(*b*) Describe any methods used to examine subgroups and interactions	
		(*c*) Explain how missing data were addressed	
		(*d*) If applicable, describe analytical methods taking account of sampling strategy	
		(*e*) Describe any sensitivity analyses	
**Results**			
Participants	13*	(a) Report numbers of individuals at each stage of study—eg numbers potentially eligible, examined for eligibility, confirmed eligible, included in the study, completing follow-up, and analysed	
		(b) Give reasons for non-participation at each stage	
		(c) Consider use of a flow diagram	
Descriptive data	14*	(a) Give characteristics of study participants (eg demographic, clinical, social) and information on exposures and potential confounders	
		(b) Indicate number of participants with missing data for each variable of interest	
Outcome data	15*	Report numbers of outcome events or summary measures	

For peer review only - http://bmjopen.bmj.com/site/about/guidelines.xhtml

Main results	16	(*a*) Give unadjusted estimates and, if applicable, confounder-adjusted estimates and their precision (eg, 95% confidence interval). Make clear which confounders were adjusted for and why they were included	
		(*b*) Report category boundaries when continuous variables were categorized	
		(*c*) If relevant, consider translating estimates of relative risk into absolute risk for a meaningful time period	
Other analyses	17	Report other analyses done—eg analyses of subgroups and interactions, and sensitivity analyses	11
**Discussion**			
Key results	18	Summarise key results with reference to study objectives	9-12
Limitations	19	Discuss limitations of the study, taking into account sources of potential bias or imprecision. Discuss both direction and magnitude of any potential bias	3,16
Interpretation	20	Give a cautious overall interpretation of results considering objectives, limitations, multiplicity of analyses, results from similar studies, and other relevant evidence	12-15
Generalisability	21	Discuss the generalisability (external validity) of the study results	12
**Other information**			
Funding	22	Give the source of funding and the role of the funders for the present study and, if applicable, for the original study on which the present article is based	22

*Give information separately for exposed and unexposed groups.

Note: An Explanation and Elaboration article discusses each checklist item and gives methodological background and published examples of transparent reporting. The STROBE checklist is best used in conjunction with this article (freely available on the Web sites of PLoS Medicine at http://www.plosmedicine.org/, Annals of Internal Medicine at http://www.annals.org/, and Epidemiology at http://www.epidem.com/). Information on the STROBE Initiative is available at www.strobe-statement.org.

For peer review only

BMJ Open

Work stress, work motivation and their effects on job satisfaction for community health workers: A cross-sectional survey in China

Journal:	*BMJ Open*
Manuscript ID:	bmjopen-2014-004897.R1
Article Type:	Research
Date Submitted by the Author:	13-May-2014
Complete List of Authors:	Li, Li; School of Public Health, Harbin Medical University, Department of Health Management<br>Hu, Hongyan; The Second Affiliated Hospital, Harbin Medical University, Research Service Office<br>Zhou, Hao; Center for Disease Control and Prevention, Department of Emergency<br>He, Changzhi; School of Public Health, Harbin Medical University, Department of Health Management<br>Fan, Lihua; School of Public Health, Harbin Medical University, Department of Health Management<br>Liu, Xinyan; School of Public Health, Harbin Medical University, Department of Health Management<br>Zhang, Zhong; School of Public Health, Harbin Medical University, Department of Health Management<br>Li, Heng; School of Public Health, Harbin Medical University, Department of Health Management<br>Sun, Tao; School of Public Health, Harbin Medical University, Department of Health Management
**Primary Subject Heading**:	Health services research
Secondary Subject Heading:	Public health
Keywords:	Human resource management < HEALTH SERVICES ADMINISTRATION & MANAGEMENT, PUBLIC HEALTH, Organisation of health services < HEALTH SERVICES ADMINISTRATION & MANAGEMENT

SCHOLARONE™
Manuscripts

Work stress, work motivation and their effects on job satisfaction for community health workers: A cross-sectional survey in China

Li Li*[1], Hongyan Hu[2], Hao Zhou[3], Changzhi He[1], Lihua Fan[1], Xinyan Liu[1], Zhong Zhang, Heng Li[1] ,Tao Sun[1]

[1] Department of Health Management, School of Public Health, Harbin Medical University, Harbin, China

[2] Research Service Office, The Second Affiliated Hospital, Harbin Medical University, Harbin, China

[3] Department of Emergency, Center for Disease Control and Prevention, Harbin, China

Correspondence to Li Li: lilihmu@gmail.com

For peer review only

Abstract

Objective: It is well documented that both work stress and work motivation are key determinants of job satisfaction. The aim of this study was to examine levels of work stress and motivation and their contribution to job satisfaction among community health workers in Heilongjiang Province, China.

Design: Cross-sectional survey.

Setting: Heilongjiang Province, China.

Participants: The participants were 930 community health workers from six cities in Heilongjiang province.

Primary and secondary outcome measures: Multistage sampling procedures were used to measure socioeconomic and demographic status, work stress, work motivation, and job satisfaction. Logistic regression analysis was performed to assess key determinants of job satisfaction.

Results: There were significant differences in some subscales of work stress and work motivation by some of socioeconomic characteristics. Levels of overall stress perception and scores on all five work stress subscales were higher in dissatisfied workers relative to satisfied workers. However, levels of overall motivation perception and scores on the career development, responsibility, and recognition motivation subscales were higher in satisfied respondents relative to dissatisfied respondents. The main determinants of job satisfaction were occupation; age; title; income;

For peer review only

the career development， and wages and benefits subscales of work stress; the recognition, responsibility and financial subscales of work motivation.

Conclusion: The findings indicated considerable room for improvement in job satisfaction among community health workers of Heilongjiang Province in China. Health care managers and policymakers should take both work stress and motivation into consideration, as two subscales of work stress and one subscale of work motivation negatively influenced job satisfaction and two subscales of work motivation positively influenced job satisfaction.

Keywords: work stress; work motivation; job satisfaction; community health service

ARTICLE SUMMARY

Article focus

▪How do the overall perception and subscales of work stress and motivation relate to levels of job satisfaction?

▪Which facets of work stress and motivation are affected by socioeconomic and demographic status?

▪How do work stress and motivation influence job satisfaction among community health workers?

Key message There is considerable room for improvement in job satisfaction among community health workers, and health care managers and policymakers should take both work stress and motivation into

For peer review only

consideration.

Strengths and limitations of this study

This study is one of the first of its kind to examine the combined effects of work stress and work motivation on job satisfaction among urban community health workers in China since the implementation of new health system reform. However, the instrument used in this study is not a commonly used international scale, there may be an inherent bias in self-report measures, and the small sample may limit the generalizability of the research findings.

For peer review only

INTRODUCTION

As the foundation of the three-tier health system in China, community health service institutions played a very important role in improving access to health care service, enhancing equity and improving health. [1-2] In 2009, the Chinese central government promulgated a new set of health system reforms and called for the development of community health services. The state established basic public health service goals, which focused on providing health education, chronic disease management, and disease prevention services for urban and rural residents. From 2009 to 2012，the number of community health service institutions increased by 6,254 and the number of visits increased by 193,949 million. Therefore, community health centers and workers thereof, are very important in the process of health system reform.

Heilongjiang Province is located in Northeast China with population of about 38.1 million. There were 410 urban community health centers (CHCs) and 366 community health stations with 13,100 health workers as of December 31, 2012.[3] On average, there were 23 and 10 medical personnel in each community health center and community health station, respectively. Since the introduction of CHCs, difficulties concerning limited resources and insufficient and poorly trained staff have been present. There were 5,416 practitioners (including assistant practitioners) in community health institutions in Heilongjiang province.[3] Based on the

For peer review only

province population and human resource planning ratios, there is an approximate shortfall of 30% in the number of general practitioners (5,416 vs. 7,620) in 2012.[4] In addition, recent reforms have expanded the scope of public health services and increased workload without equivalent increases in staffing.[5,6]

In some CHCs, general practitioners, public health physicians, and nurses have been working in teams, providing medical and basic public health services to community residents, both in the centers and during home visits. With a late start, many of these practitioners were initially hospital-based specialists, and majority of public health physicians did not have a public health background. To improve skills and knowledge, continuing medical education was compulsory and no fewer than 25 credit points were required per year. Other challenges found in the CHCs were lower wages and fewer title promotion opportunities relative to general hospitals. Limited resources and a shortage of skilled health workers created very tight bottlenecks in the provision of services, which led to many community health workers experiencing work-related stress and low motivation for work, in addition to receiving low salaries and restricted opportunities for promotion.[7-8] Many studies have shown that work stress and work motivation can greatly affect job satisfaction and, in turn, the quality and delivery of health care. However, few studies have focused on work stress and motivation and their effects on job

For peer review only

satisfaction among Chinese community health workers since the implementation of the new health system reform policy.

Work stress is of great concern to managers, employees, and other stakeholders in organizations. It is a complex phenomenon and has a multitude of definitions in a variety of theoretical models.[9] According to Lazarus and Folkman's cognitive theory of stress and coping, work stress was defined as the interaction between the individual and the environment.[10] This theory suggested that when demands from the environment exceed the available resources, the result was either stress or coping, depending on the individual's appraisal of the stressors. Karasek's demand-control model assumed that psychological strain resulted from the joint effects of work demands and the degree of decision-making freedom available to workers facing the demands.[11] The effort-reward imbalance model proposed that work stress resulted from a mismatch between high commitment and effort at work and low rewards, including salary, recognition, and career promotion.[12] Nakasis and Ouzouni defined work stress as the harmful physical and emotional responses that occur when job requirements do not match workers' capabilities, resources, and needs.[13] In general, a greater imbalance between demands and individual abilities will result in greater stress.[14] Riggio classified work stress into work task stress and work role stress.[15] Cooper and Marshall's model of job stress proposed that intrinsic requirements of the job, role within the

For peer review only

organization, career development, organizational structure and climate, and relationships at work constituted the domain of work-related stress within an organization.[16] In our study, five subscales of work stress were named based on this model. Existing research has identified heavy workload, insufficient resources, work relations, lack of professional respect, and lack of promotion opportunities as possibly the most salient work stressors for community health workers.[17-19] Long-term stress may not only be harmful to the health workers themselves but may also affect community health service centers through employee dissatisfaction, burnout, poor performance, or turnover intention.[20,21-24] Therefore, it is important to reduce work stress.

Work motivation can be defined as the degree of an individual's willingness to exert and maintain an effort towards attaining organizational goals.[25] It reflected the interactions between workers and their work environments. Nahavandi and Malekzadeh believed that motivation represented a stable mind, aspiration, or interest within the individual and can translate into action.[26] Motivation theory examined the process of motivation and explained why people at work behave the way they do in terms of efforts. Building on Vroom's expectancy-valence theory of motivation, Porter and Lawler proposed a model of intrinsic and extrinsic work motivation.[27,28] This model suggested that intrinsic and extrinsic rewards were additive, and accounted for total job satisfaction.

For peer review only

Intrinsic motivation refers to doing something for the inherent satisfaction involved and is highly autonomous (i.e., self-regulated). In contrast, extrinsic motivation means doing something in order to obtain a separable outcome (i.e. tangible or verbal rewards).[29,30] Peters identified job content and work environment, extrinsic benefits, autonomy and security, and transparency as factors in work motivation for health workers using factor analysis.[31] Patrick and Wilbroad developed a tool to measure health worker motivation and revealed that organizational commitment, conscientiousness, intrinsic job satisfaction, timeliness and attendance were the major determinants of higher motivation.[32-33] Tribolet explored the relationship between intrinsic and extrinsic motivation.[34] Hoonakker found that nurses appreciated challenges and opportunities for new learning and teamwork.[35] Pool explored the significant positive association between work motivation and job satisfaction, whereas Stringer found that intrinsic motivation was positively associated, and extrinsic motivation negatively associated with job satisfaction.[36-37]

In China, previous studies have reported that poor competency and excessive workload were key work stressors among community health workers.[7,19] Shi suggested that policymakers should focus on training and educational opportunities for primary care workers and consider ways to reduce workload stress and improve salaries. [38] Hung identified professional development, training opportunities, living environment,

For peer review only

benefits, and working conditions as the most important motivating factors for primary care providers in China.[39] Ge analyzed the relationship between work stress and job satisfaction among Chinese community health workers and reported that a degree of freedom in decision making and good workplace relationships were positive predictors of job satisfaction.[40] Chen investigated relationships between work motivation, work stress and job satisfaction in cross-strait employees in Taiwan and mainland China.[41]

The present study focused on the major factors affecting work stress and motivation identified in previous research and provided an overview of community health workers' perspectives of work stress and motivation factors.[16, 42-44] The purpose of this study was to assess the predictors of job satisfaction among community health workers in one Chinese province. A cross-sectional survey was conducted to measure levels of work stress, work motivation and job satisfaction. The key predictors of job satisfaction for community health workers were assessed with special attention devoted to work stress and motivation.

METHODS

Sample

A cross-sectional survey of community health workers was conducted from March 1 to October 31, 2013 in Heilongjiang Province, China.

For peer review only

Based on the literature about community health services in China, a multistage, stratified sampling design was employed to ensure that study data were provincially representative.[7,40] First, six cities (Harbin, Qiqihar, Suihua, Jiamusi, Qitaihe, and Heihe) were selected based on GDP figures and three levels of the development of the community health service. Second, 15 community health centers were randomly selected from each city. On average, there were 22 medical personnel in each of the selected community health centers. Third, 60% of general practitioners, public health physicians, nurses and other health technical staff in each center were chosen randomly, with the exception of those who were absent. The research team invited all the selected staff members to participate in the study. The questionnaire included a cover page explaining the purposes and procedures of the study. The data were collected anonymously and the respondents completed the survey questionnaires privately to ensure confidentiality. Respondents were assured that participation in the survey was voluntary, and the return of questionnaires represented informed consent. The research staff stayed at the community health center and answered respondents' questions during the process of survey completion. Respondents were able to choose the best time to complete the questionnaire, such as when they were not busy or their offices were quiet. Most completed questionnaires were collected on site by the investigator on the day of the visit. If some respondents did

For peer review only

not finish that day, investigators set a date to retrieve the questionnaires. Respondents were asked to seal the completed questionnaires into individual envelopes provided by the research team. The questionnaire was relatively brief and no private personal information was collected. There were 980 questionnaires delivered to community health workers, all of which were returned. However, 50 (5.1%) were incomplete or even blank, which left 930 valid questionnaires. This study was approved by Medical Ethic Committee of Harbin Medical University.

Assessment tools

In the present study, Porter and Lawler's intrinsic and extrinsic motivation model, and Voom's expectancy-valence motivation theory were used to analyze the relationship between work motivation and job satisfaction. Lazarus and Folkman's cognitive theory of stress and coping, and Karasek's demand-control model were used to analyze the relationship between work stress and job satisfaction.[10-11]

The study instrument was part of a self-administered questionnaire composed of four sections. Section 1 focused on respondents' socioeconomic and demographic status.

Section 2 assessed work stress. Thirty items related to work stress were developed through intensive qualitative interviews with policymakers, health care managers and community health workers, a

For peer review only

review of the literature, and an initial pilot study.[16,42] Then factor analysis, which was not discussed in this paper, yielded a five-subscale structure that comprised a total of 26 items. The five-subscale solution accounted for 69.43% of the overall variance, and was found to be internally consistent (overall Cronbach's α=0.87). Based on Cooper and Marshall's model of job stress, these five subscales of work stress were named work task and role, career development, wages and benefits, workplace relationships, and organizational structure and climate stress.[16] They individually accounted for 16.05%, 25.10%, 12.00%, 9.08% and 7.20% of the overall variance, respectively, and the Cronbach's Alpha within individual subscale ranged from 0.85 to 0.90. Respondents were asked to rate their perception of work stress on each item based on a 5-point Likert scale, very less stressful (1), less stressful (2), average (3), stressful (4) and very stressful (5). The Cronbach's alpha value for this study was 0.87.

Section 3 assessed work motivation. Twenty-one items were developed based on previous research, panel discussions, and an initial pilot study.[43-45] Then 3 items were deleted and the 18 retained items were divided into four subscales by factor analysis, which was not discussed in this paper. The four-subscale solution accounted for 65.10% of the overall variance, and was found to be internally consistent (overall Cronbach's α=0.75). The subscales were renamed based on the conceptual meaning

For peer review only

of the items and comprised: career development, recognition, responsibility, and financial motivation. They individually accounted for 21.20%, 19.40%, 14.60% and 9.90% of the overall variance, and the Cronbach's Alpha within individual subscale ranged from 0.82 to 0.89. According to Porter and Lawler's intrinsic and extrinsic motivation model, we defined career development and financial motivation as extrinsic motivation, and recognition and responsibility motivation as intrinsic motivation.[28,44] Respondents were asked to rate their motivation intensity on each item based on a 5-point Likert scale, very less strong (1), less strong (2), average (3), strong (4) and very strong (5).

Section 4 assessed job satisfaction. In this study, a single-item measure was used to measure overall job satisfaction.[46] Respondents were asked to indicate their level of job satisfaction on a 4-point Likert scale, strongly dissatisfied (1), dissatisfied (2), satisfied (3) and dissatisfied (4). During the process of data analysis, strongly satisfied and satisfied were coded as 1, while strongly dissatisfied and dissatisfied were coded as 0.

Data analysis

Survey results were analyzed using SPSS 17.0. Descriptive analyses included frequencies and percentages for categorical variables and means and standard deviations (SDs) for continuous variables. Mean differences

For peer review only

were examined using t-tests and ANOVAs for relevant subgroups. We used logistic regression to measure the key predictors of job satisfaction because the dependent variable (job satisfaction) was a binary variable, which made linear regression unsuitable.

RESULTS

Socioeconomic and demographic status of respondents

Socioeconomic and demographic status of the sample were shown in Table 1. A majority of the participants were female (74.6%). General practitioners accounted for 36% of community health workers surveyed, followed by nurses (28.8%), public health physician (19.1%). In this survey, only 18.6% of them had senior professional titles and less than half (40.2%) of them had bachelor degree or higher. Only 19.6% of them had monthly incomes of more than 3,000 RMB (where \$1.00US=6.23RMB in 2012). Nearly ninety percent of respondents worked more than 40 hours per week.

Work stress and motivation according to socioeconomic and demographic factors

Results of variance analysis and further multiple comparison t-tests were shown in Table 1. There were significant differences in scores for all of the five subscales of work stress according to occupation ($p < 0.01$) and gender ($p < 0.05$), with general practitioners and men showing higher

For peer review only

levels of work stress.

Scores for the wages and benefits subscale of work stress differed significantly according to educational background ($p < 0.05$) and income ($p < 0.05$). Mid-level professionals reported significantly higher levels of stress on the work task and role subscale ($p < 0.01$) and in workplace relationships ($p < 0.05$). Participants aged 35–44 and 45–54 years reported significantly higher levels of stress on the work task and role subscale ($p < 0.01$).

The male had significant higher level recognition and financial motivation ($p<0.05$). Younger workers (<25) had significantly higher level of recognition motivation ($p<0.05$) and responsibility motivation ($p<0.05$). A higher level of recognition motivation was expressed by general practitioners ($p<0.05$).

There were no significant differences in any of the four work motivation subscale scores according to educational background, professional title, or income. Men reported significantly higher levels of recognition and financial motivation ($p < 0.05$). Younger workers (<25) reported significantly higher levels of recognition ($p < 0.05$) and responsibility motivation ($p < 0.05$). General practitioners reported higher levels of recognition motivation ($p < 0.05$).

Insert Table 1 here

For peer review only - http://bmjopen.bmj.com/site/about/guidelines.xhtml

Levels of work stress, work motivation, and job satisfaction

The mean score for overall perception of work stress was 3.11, which is slightly higher than the mid-point of 3 (Table 2). Wages and benefits (3.60) subscale of work stress ranked in the highest position, followed by work task and role (3.31), career development (2.96), organizational structure and climate (2.90), and relationships (2.75) (F=154.9，p<0.001). Statistically significant differences were noted in overall perception of stress and scores on all five work stress subscales between satisfied and dissatisfied respondents; those who were dissatisfied reported higher levels of work stress (p < 0.001).

Career development motivation was rated the highest level, followed by financial, recognition and responsibility motivation (F=202.6，p<0.001). Levels of overall perception of work motivation and all subscales with the exception of financial motivation were significantly different between the satisfied and dissatisfied groups of respondents, and the satisfied workers had higher levels of work motivation (p<0.01).

In respect to motivation, career development was rated highest, followed by financial, recognition, and responsibility motivation (F = 202.6，p < 0.001). Levels of overall perception of motivation and scores on all work motivation subscales, with the exception of financial motivation, differed significantly between the satisfied and dissatisfied

For peer review only

respondents, and the satisfied workers reported higher levels of work motivation ($p < 0.01$).

Insert Table 2 here

Predictors of job satisfaction

In this study, 61.3% of respondents were satisfied with their jobs. Table 3 presented results of a logistic regression model that examined the key predictors of job satisfaction, with special attention devoted to work stress and work motivation.

Only a few demographic characteristics were predictors of job satisfaction. We found that when scores on the career development and wages and benefits subscales of work stress increased by one grade, job satisfaction decreased by 32% (odds ratio [OR] = 0.68, $p < 0.05$) and 37% (OR = 0.63, $p < 0.01$), respectively. When financial motivation increased by one grade, job satisfaction decreased by 28% (OR = 0.72, $p < 0.01$), and when recognition motivation and responsibility motivation increased by one grade, job satisfaction increased 1.86 (OR = 2.86, $p < 0.01$) and 0.36 times (OR = 1.36, $p < 0.05$), respectively. Compared with nurses, general practitioners (OR = 0.56, $p < 0.01$) and public health physicians (OR = 0.42, $p < 0.05$) reported lower job satisfaction, while other technical staff (OR = 1.89) reported higher job satisfaction. Workers

For peer review only

with no title (OR = 7.02, $p < 0.05$) were more satisfied than workers with a senior title.

Insert Table 3 here

Discussion

Job satisfaction in community health workers is important for the sustainable development of basic healthcare in China, but health policymakers and managers have neglected it for a long time.[47] This study was one of the first of its kind to examine the level of work stress and work motivation and their combined effects on job satisfaction among urban community health workers in China since the implementation of new health system reform.

Results indicated that the wages and benefits subscale of stress ranked highest, followed by the work task and role subscale. Similarly, previous research related to work stress found that low salary, heavy workload, and few promotion opportunities were the most frequently cited workplace stressors.[49,50] Several reasons may have contributed to these findings. In Heilongjiang Province, the average annual income of health service personnel in urban hospitals was 52,564 RMB ($1.00US = 6.23RMB) in 2012. In this study, 80.4% of the respondents' annual incomes were lower than 36,000 RMB. These low salaries for community

For peer review only

health workers increased their wages and benefits stress.[48] In the meanwhile, based on the province population and human resource planning ratios, there is an approximate shortfall of 30% in the number of general practitioners in 2012.[4] And the recent reforms have expanded the scope of public health services and increased workload without equivalent increases in staffing.[5,6]

Unfortunately, the present study found that scores on the career development, and wages and benefits subscales of work stress were negatively associated with job satisfaction. These findings were consistent with previous studies in which workers were likely to report low job satisfaction if they did not receive promotion and advancement opportunities or adequate salaries.[22,33,52]

With regard to work motivation, results showed the career development and financial subscales of work motivation ranked first and second respectively. Consistent with Hung and Hou's study, which found income, benefits, and professional development were the most important motivating factors among community health workers in China.[39,51]

In this study, we defined career development and financial motivation as extrinsic motivation and recognition and responsibility motivation as intrinsic motivation based on the literature.[37,53] Results reported that the recognition and responsibility subscales of work motivation were positive predictors of job satisfaction, and financial

For peer review only

motivation was a negative predictor. This was consistent with the "crowding-in" effect, which proposes that intrinsic motivation increases job satisfaction, whereas extrinsic motivation decreases job satisfaction.[54] It should be noted that in this study, the level of extrinsic motivation was higher than that of intrinsic motivation.

These findings have significant implications for managers of community health centers and policymakers in their efforts to improve workers' job satisfaction. First, policymakers should take measures to improve community health workers' salaries. In China, basic public health services are funded by the government and provided by community health workers without cost to residents. If health workers are dissatisfied with their salaries, they may prefer to work for profit-making medical services instead of nonprofit public health services. In the meanwhile, managers should implement appropriate performance salary distribution system to arouse the enthusiasms of the staff and reduce their financial stress. Second, policymakers should focus on appropriate promotion policies for community health workers. At present, it was difficult for community health workers to get title promotion, for there were limit promotion quotas for CHCs every year in Heilongjiang Province and our study found only 18.6% of respondents had senior professional title. Third, the managers should provide and support their workers to attend training or continuing education. Fourth, managers and

policymakers should take measures to inspire intrinsic motivation in workers. Becchetti proposed that when workers do not work for financial incentives, they may find satisfaction irrespective of their salaries, even if the financial incentive is kept to a minimum, and may therefore be satisfied with their jobs.[53] Therefore, managers and policymakers should introduce more incentives to encourage community health workers to work for responsibility or recognition.

As some subscales of work stress and work motivation can influence job satisfaction either positively or negatively, we examined levels of work stress and motivation according to demographic characteristics and found that policymakers and managers should pay more attention to three types of workers. The first group of workers included those aged between 35 and 54 years (35–44 and 45–54 age groups), who reported higher levels of stress on the work task and role subscale and lower levels of intrinsic motivation. Similar results have been reported; in Qu's study, community health workers in mid-level age groups were significantly more stressed than those in the youngest age group in one province of China.[55] This could be related to workload or difficulty and complexity of the work task, which is usually greater for 35- to 54-year-old workers, as they are the backbone of community health services. Men form the second group that requires attention. In our study, men's scores on all of the work stress subscales tended to be higher than women's, and men

For peer review only

reported higher levels of financial motivation.[56] However, another Chinese study of primary health workers found no differences in financial motivation.[51] David found that women reported more stress in the financial rewards and role ambiguity subscales.[57] The final group of workers identified as requiring attention consists of general practitioners, who experienced the highest stress according to all five work stress subscales and reported the highest career development motivation. General practitioners in community health centers face more difficult and complicated tasks and types of medical risk than other health care workers do, and they receive lower salaries and fewer promotion opportunities than their counterparts in general hospitals.

Limitations of this study

The findings in this study should be viewed in light of four key limitations. First，this study was based on a small sample of community health workers, which may limit the generalizability of the research findings. Based on the literature about community health services in China, a multistage, stratified sampling design was employed to ensure that study data were provincially representative. [7,40] A multistage, stratified sampling design was used to ensure that study data were provincially representative. Six sample cities were selected to account for the variability in regional per capita gross domestic product, and the

For peer review only

levels of healthcare development and 15 CHCs in each city were selected randomly. On average, there are 23 medical technical personnel in a community health center in Heilongjiang province and there were approximately 22 health workers in each of the community health centers in our study. In addition, the proportions of general practitioners, public health physicians, nurses, and other medical technical personnel in this study were close to the proportions found in the province as a whole.[3] Consequently, this sample was representative of Heilongjiang community health service providers, thereby enhancing the potential for generalization of the study findings. Second, the instrument for assess the work stress and work motivation was developed from earlier study and discussed with experts, while not an international commonly scale. Third, we used a cross-sectional survey, which may limit our ability to identify causal relationships between work stress and motivation and job satisfaction. Fourth, the measurements were conducted by self-administrated method and respondents' cognition can be affected by emotions at that point in time. So the common method bias and self-administrated bias might affect the results.

Conclusion

It is important for healthcare managers to improve the job satisfaction of health workers in low-resource settings. In this study, we examined levels

For peer review only

of work stress and motivation according to demographic characteristics and in respect to levels of job satisfaction; additionally, the key predictors of job satisfaction were identified using logistic regression analysis. The results indicated that community health workers rated wages and benefits highest among five subscales of work stress, and workers' extrinsic motivation was higher than their intrinsic motivation. The career development，and wages and benefits subscales of work stress and financial motivation were significant negative predictors of job satisfaction, whereas the recognition and responsibility subscales of work motivation were significant positive determinants.

Our findings suggest that there is considerable room for improvement in the job satisfaction of community health workers in Heilongjiang Province, and health care managers and policymakers should take both work stress and work motivation into consideration. First, they should pay more attention to three types of worker, as these particular groups reported higher work stress and extrinsic motivation. Second, they should take a variety of measures to reduce career development, and wage and benefits stress, as they were negative determinants of job satisfaction. Third, it is important for managers and policymakers to inspire workers' intrinsic motivation, as it can have a positive influence on job satisfaction.

For peer review only

Acknowledgements We are thankful to all the community health workers who participated in the study. We are also grateful to Yin Li, Xingsan Li, Zhuang Wang and Hongjuan Wei, who have worked closely with the team to ensure the field survey is successfully implemented.

Funding This study was funded by the National Science Foundation of China (NSFC), Contract No.71203050/G0308, Contract No.71073034 and was supported by Young Seed Foundation of Public Health College of Harbin Medical University. The opinions expressed herein are the authors' and do not necessarily reflect the views of NSFC and the survey was conducted independently by researchers from Harbin Medical University.

Contributors

LiLi was responsible for the study design, data analysis and the drafting and revising of the manuscript. HongyanHu and ChangzhiHe, who contributed equally as the first author to this article, were responsible for study design, data collection and data analysis. HaoZzhou and ZhongZ hang provided statistical expertise. XinyanLiu, TaoSun and HengLi performed data collection and technical support. LihuaFan provided administrative support. All authors read and approved the final manuscript.

For peer review only

Competing interests None.

Data Sharing Statement: The factor analysis of work stress and work motivation are available to Li Li at lilihmu@gmail.com.

For peer review only

References

1. Starfield B, Shi LY, Macinko J. Contribution of primary care to health systems and health. Milbank Q 2005;83: 457–502.
2. Hung LM, Rane S, Tsai J, et al. Advancing primary care to promote equitable health: implications for China. Int J Equity Healt 2012;11:2.
3. Heilongjiang Provincial Bureau. Heilongjiang Health Statistical Yearbook 2012. Harbin, China: Heilongjiang Provincial Press 2013.
4. State Commission Office for Public Sector Reform. (2006). Notice on Issuing the Instruction Opinions on Organization Structure and Staffing of Urban Community Health Service. Beijing, China. http://www.gov.cn/zwgk/2006-09/04/content_377067.htm (accessed on 4 September 2006).
5. MOH (Ministry of Health of China) Chinese Health statistics in 2010. Peking Union Medical College Press 2010.
6. MOH (Ministry of Health of China) (2012) Chinese Health statistics in 2010. Peking Union Medical College Press.

For peer review only

7. Zhou W, Dong YM, Lin XZ, et al. Community health service capacity in China: a survey in three municipalities. J Eval Clin Pract 2013;19(1):167–17.
8. Li L, Zhou H, Yao GF, et al. Analysis on working motivation of community health workers in Harbin, China. Medicine and Society (in Chinese) 2013;7:40-43.
9. Clegg A. Occupational stress in nursing: a review of the literature. J Nurs Manage 2001; 9:101–106.
10. Lazarus RS. & Folkman S. Stress, Appraisal and Coping. Springer, New York, NY.1984.
11. Karasek RA. Job demands, job decision latitude, and mental strain: implications for job re-design. Administrative Science Quarterly 1979; 24(2): 285–308.
12. Siegrist J. Adverse health effects of high-effort/low-reward conditions. J Occup Health Psychol 1996; 1:27–41.
13. Nakasis K., Ouzouni C. Factors influencing stress and job satisfaction of nurses working in psychiatric units: a research review. Heal Sci J 2008;2 (4): 183–195.
14. Jamal M. Burnout among Canadian and Chinese employees: a cross-cultural study. European Management Review 2005;2(3):224–230.

For peer review only

15. Riggio R. Introduction to industrial organizational psychology. New Jersey: Pearson Education, Inc. 2003.

16. Cooper CL, Marshall J. Occupational sources of stress: a review of the literature relating to coronary heart disease and mental ill health. J Occup Psychol 1976; 49:11-28.

17. Kim HJ. Work Stress and Job Satisfaction of Community Mental Health Nurses in South Korea: A Qualitative Content Analysis. J Korean Acad Psychiatr Ment Health Nurs 2013;22(4):295-306.

18. Johnson SJ, O'Connor EM, Jacobs S, et al. The relationships among work stress, strain and self-reported errors in UK community pharmacy. Research in Social and Administrative Pharmacy. 2014;1.

19. Qu NQ, Zhou H, Fan LH, et al. Analysis on work stress and occupational burnout among community health service staff in Harbin. Medicine and Society 2013;26(9):87-89.

20. Chen XJ, Tan XR, Li LP. Health Problem and Occupational Stress among Chinese Doctors. Chinese Medicine 2013;4: 1-6.

21. Nabirye RC, Brown KC, Pryor ER et al. Occupational stress, job satisfaction and job performance among hospital nurses in Kampala, Uganda. J Nurs Manage 2011; 19:760–768.

For peer review only

22. Voltmer E, Rosta J, Johannes Siegrist, et al. Job stress and job satisfaction of physicians in private practice: comparison of German and Norwegian physicians. Int Arch Occ Env Hea 2012;85, (7):819-828.

23. Saijo Y, Chiba S, Yoshioka E, et al. Job stress and burnout among urban and rural hospital physicians in Japan. Aust J Rural Health 2013;21(4): 225–231.

24. Sun Y, Luo ZN, Fang PQ. Factors Influencing the Turnover Intention of Chinese Community Health Service Workers Based on the Investigation Results of Five Provinces. J Commun Health 2013;38(6):1058-1066.

25. Franco LM, Bennett S, Kanfer R, et al. Determinants and consequences of health worker motivation in hospitals in Jordan and Georgia. Soc Sci Med 2004;58:343-355

26. Kanfer R. Measuring health worker motivation in developing countries. Partnerships for Health Reform Project, Major Applied Research Working Paper 1999.

27. Vroom, V. H. Work and motivation. New York: Wiley.1964.

28. Porter LW, Lawler EE. Managerial attitudes and performance. Homewood, IL: Irwin-Dorsey 1968.

29. Ryan RM, Deci EL. Self-determination theory and the facilitation of intrinsic motivation, social development, and

For peer review only

wellbeing. Am Psychol 2000; 55(1): 68–77.

30. Ryan RM, Deci EL. Self-regulation and the problem of human autonomy: Does psychology need choice, self-determination, and will? J Pers 2006; 74(6):1558–1585.

31. Peters DH, Chakraborty S, Mahapatra P, et al. Job satisfaction and motivation of health workers in public and private sectors: cross-sectional analysis from two Indian states. Hum Resour Health. 2010;8:27.

32. Patrick MM, Duane B, Lucy G, et al. Developing a tool to measure health worker motivation in district hospitals in Kenya. Hum Resour Health 2009; 7:40.

33. Wilbroad M, Helen A, Virginia B, et al. Measuring health workers' motivation in rural health facilities: baseline results from three study districts in Zambia. Hum Resour Health 2013;11:8.

34. Tribolet WC. The relationship between intrinsic and extrinsic motivation and organizational commitment: a study in a European environment [D]. Nova Southeastern Univ 2004.

35. Hoonakker PL, Carayon P, McGuire K, et al. Motivation and job satisfaction of Tele-ICU nurses. J Crit Care 2013;28:315.e13 -315.e21.

For peer review only

36. Pool SW. The relationship of job satisfaction with substitutes of leadership, leadership behavior, and work motivation. J Psychol 1997;131(3):271-83.

37. Stringer C, Jeni D, Theivananthampillai. Motivation, pay satisfaction, and job satisfaction of front-line employees. Qual Res Acco Man 2011; 8(2):161-179.

38. Shi L, Hung LM, Song K, et al. Chinese primary care physicians and work attitudes. Int J Health Serv 2013;43(1):167–181.

39. Hung LM, Shi L, Wang H, et al. Chinese primary care providers and motivating factors on performance. Fam Prac 2013; 30:576–586.

40. Ge C, Fu J, Chang Y, et al. Factors associated with job satisfaction among Chinese community health workers: a cross-sectional study. BMC Public Health, 2011;24(11):884.

41. Chen TL, Huang MY, Su TH. Work motivation, work stress, and job satisfaction in between Taiwan and China-An empirical study. World Acad Sci, Eng Tech 2012; 68:1446-1450.

42. Ajayi MP., Abimbola OH. Job satisfaction, organizational stress and employee performance: a study of NAPIMS. Ife

For peer review only

Psychol 2013;21:75-82.

43. Wayne SJ, Liden RC. Effects of impression management on performance ratings: a longitudinal study. Acad Manage J 1995;38:232-260.

44. Gagne M, Deci EL. Self-determination theory and work motivation. J Organ Behav 2005;26:331-362.

45. Dieleman M, Cuong PV, Anh, LV, et al. Identifying factors for job motivation of rural health workers in North Vietnam. Hum Resour Health 2003;1:10.

46. Wanous JP, Reichers AE, Hudy MJ. Overall job satisfaction: how good are single-item measures? J Appl Psychol 1997;82:247–52.

47. Chen J, Dong JQ, Ding J, et al. Analysis on satisfaction of professionals in community health service institutions held by different levels of hospital. Chin Gen Pract 2008;4(11): 631-634. (In Chinese)

48. National Bureau of Statistics. China Statistic Yearbook 2013. Beijing ,China: China Sta Press. 2013.

49. Ding H, Sun X, Chang WW, et al. A comparison of job satisfaction of community health workers before and after local comprehensive medical care reform: a typical field investigation in central China. Plos One 2013;8(9):1-5.

For peer review only

50. Han JF, Li XH. An analysis on the job stressors of community health nurses.Chinese Nursing Management. 2007;7(5):45-48. (in Chinese)

51. Hou ZH, Meng QY, Yuan BB et al. Motivation preferences of general practices in rural China. Chinese Journal of Health Policy, 2010;13(10):18-22.

52. Lephalala RP. Factors influencing nurses job satisfaction in selected private hospitals in England. Curationis 2008;31(3): 60–69.

53. Becchetti L, Castriota S, Tortia EC. Productivity, wages and intrinsic motivations. Sma Bus Eco 2012;41:379–399.

54. Frey BS. On the relationship between intrinsic and extrinsic work motivation. Int J Ind Organ 1997;15(4),427-439.

55. Qu JW. Investigation and research on job stress and degree of loyalty in community health workers in Liaoning Province (M). Shenyang: Chinese Medical University 2013.

56. Malik AA, Yamamoto SS, Souares A, et al. Motivational determinants among physicians in Lahore Pakistan. BMC Health Serv Res 2010;10:201.

57. David PH, Srinika DJ. Gender differences in work stress among clinical social workers. J Soc Serv Res 2008;10:18.

For peer review only

For peer review only

Table 1 Facets of work stress and work motivation by socio-economic and demographic status for respondents

			Work stress					Work motivation			
	N	%	work task and role	career development	wages and benefits	Workplace relationships	organizational structure and climate	recognition	career development	responsibility	Finance
**Occupation**											
**General practitioner**	335	36.0	3.53	3.17	3.78	2.90	3.14	3.61	4.20	3.44	4.15
**Public health physician**	178	19.1	3.20	2.89	3.70	2.63	2.96	3.57	4.11	3.67	4.06
**Nurse**	267	28.8	3.24	2.95	3.54	2.76	2.78	3.53	4.05	3.39	4.01
**Other**	150	16.1	3.09	2.79	3.45	2.65	2.84	3.59	4.11	3.40	4.03
**F**			6.91**	4.97**	3.45**	3.05**	6.25**	0.66	2.31*	1.96	0.99
**Sex**											
**Male**	236	25.4	3.44	3.10	3.77	2.88	3.12	3.71	4.18	3.50	4.19
**Female**	694	74.6	3.27	2.93	3.56	2.72	2.85	3.56	4.12	3.43	4.03
**F**			2.50*	2.27*	2.60*	2.51*	4.09*	2.36*	1.23	1.04	2.39*
**Educational background**											
**High school or below**	110	11.8	3.18	2.90	3.36	2.81	2.72	3.57	4.13	3.42	4.05
**Junior college**	446	48.0	3.28	2.94	3.61	2.74	2.86	3.57	4.14	3.49	4.02
**College and above**	374	40.2	3.36	3.00	3.65	2.73	3.16	3.60	4.11	3.39	4.11
**F**			2.30	0.66	4.21*	0.45	4.02*	0.13	0.24	1.53	1.33
**Age in years**											
**<25**	78	8.4	3.08	2.81	3.45	2.60	2.77	3.80	4.23	3.60	3.92
**25-34**	258	27.7	3.21	2.94	3.63	2.72	2.91	3.65	4.11	3.48	4.17

For peer review only - http://bmjopen.bmj.com/site/about/guidelines.xhtml

35-44	329	35.4	3.36	2.98	3.55	2.78	2.88	3.52	4.13	3.35	4.03
45-54	234	25.2	3.43	3.02	3.69	2.79	2.94	3.53	4.13	3.51	4.04
≥55	31	3.3	3.12	2.88	3.54	2.71	2.93	3.48	3.98	3.28	3.91
F			4.71**	1.01	1.36	1.12	0.83	2.89*	1.83	2.86*	2.39
**Title**											
Senior title	42	4.5	3.12	3.11	3.38	2.69	2.73	3.37	3.97	3.55	3.96
Vice-senior title	131	14.1	3.32	2.92	3.65	2.63	2.93	3.46	4.05	3.25	4.03
Middle title	399	42.9	3.43	3.03	3.69	2.85	2.94	3.56	4.16	3.44	4.06
Primary title	299	32.2	3.20	2.93	3.54	2.72	2.87	3.62	4.12	3.49	4.08
No title	59	6.3	3.23	2.86	3.48	2.58	2.89	3.73	4.16	3.49	4.04
F			3.96**	1.07	1.71	3.04*	0.59	1.73	0.98	2.13	0.16
**Monthly income （RMB）**											
<2000	361	38.9	3.24	2.95	3.69	2.76	2.90	3.61	4.15	3.49	4.09
2000-2999	386	41.5	3.32	2.96	3.61	2.75	2.88	3.59	4.13	3.40	4.06
3000-3999	139	14.9	3.43	2.97	3.44	2.68	2.96	3.52	4.02	3.43	3.97
≥4000	44	4.7	3.39	3.03	3.21	2.93	2.78	3.44	4.27	3.53	4.28
F			2.11	0.99	3.14*	2.11	0.99	0.54	1.87	0.86	1.36
**Working hours (per week)**											
<40 小时	110	11.8	3.27	2.82	3.52	2.82	2.94	2.96	4.14	3.56	3.91
40-47 小时	509	54.7	3.26	2.73	3.59	2.73	2.95	2.87	4.10	3.42	4.06
48-55 小时	250	26.9	3.36	2.71	3.62	2.71	2.93	2.89	4.19	3.46	4.12
≥56 小时	61	6.6	3.52	2.93	3.75	2.93	3.36	3.13	4.16	3.43	4.14
F			0.06	0.20	0.48	0.20	0.01*	0.11	0.39	0.44	0.13

*p<0.05 **p<0.01

For peer review only

Table 2 Mean scores of the overall perception and subscales of work stress and work motivation in respect to the level of job satisfaction

	Mean±SD Total (n=930)	Level of job satisfaction Satisfied (n=570, 61.3%)	Dissatisfied (n=360, 38.7%)	P
**Work stress**				
**Overall perception** ※	3.11±0.68	2.95±0.68	3.37±0.60	P=0.000
**work task and role¶**	3.31±0.81	3.18±0.82	3.52±0.76	P=0.000
**career development¶**	2.96±0.87	2.79±0.85	3.22±0.83	P=0.000
**Wages and benefits ¶**	3.60±0.95	3.38±0.94	3.95±0.85	P=0.000
**Workplace relationships¶**	2.75±0.79	2.61±0.79	2.96±0.74	P=0.000
**organizational structure and climate¶**	2.90±0.79	2.74±0.79	3.15±0.71	P=0.000
**Work motivation**				
**Overall perception**◎	3.80±0.55	3.86±0.55	3.70±0.55	P=0.000
**Career development**†	4.13±0.57	4.24±0.51	3.95±0.62	P=0.000
**Recognition**†	3.58±0.77	3.66±0.77	3.45±0.77	P=0.000
**Responsibility**†	3.45±0.77	3.53±0.77	3.32±3.52	P=0.000
**Financial**†	4.06±0.79	4.02±0.79	4.12±0.80	P=0.295

※ Mean score of overall perception of work stress was calculated for each respondent by adding the value of each item of work stress and then divided by the numbers of all item.

◎Mean score of overall perception of work motivation was calculated for each respondent by adding the value of each item of work motivation and then divided by the numbers of the item.

¶Mean score of each subscale of work stress was calculated for each respondent by adding the value of each item belongs to the subscale of work stress and then divided by the numbers of the item.

†Mean score of each subscale of work motivation was calculated for each respondent by adding the value of each item belongs to the subscale of work motivation and then divided by the numbers of the item.

For peer review only

Table 3 The logistic regression analysis for job satisfaction※

		Odds Ratio	95% CI
**Occupation (Reference : nurse)**	General practitioner	0.56**	0.38-0.81
	Public health physician	0.42*	0.20-0.87
	Other technical staff	1.89*	1.04-3.44
**Sex (Reference :male)**	Female	1.27	0.83-1.95
**Educational background (Reference :High school or below)**	Junior college	0.76	0.43-1.34
	College and above	0.75	0.41-1.40
**Age in years (Reference : <25)**	25-34	0.60	0.30-1.21
	35-44	1.10	0.51-2.42
	45-54	1.04	0.45-2.35
	≥55	8.53**	1.86-39.01
**Title (Reference : senior title)**	Vice-senior title	1.86	0.476-7.29
	Middle title	2.57	0.67-9.78
	Primary title	3.84	0.96-15.39
	No title	7.02*	1.53-32.12
**Monthly income in RMB (Reference : <2000)**	2000-2999	0.50	0.26-0.98
	3000-3999	0.99	0.64-1.52
	≥4000	1.30	0.86-1.97
**Weekly hours worked (Reference :<40)**	40-47	0.90	0.59-1.37
	48-55	1.07	0.67-1.70
	≥56	1.20	0.62-2.33
**Work stress**	work task and role	0.98	0.74-1.300
	career development	0.68*	0.49-0.94
	wages and benefits	0.63**	0.50-0.79
	Workplace relationships	0.80	0.59-1.09
	Organizational structure and climate	0.97	0.71-1.33
**Work motivation**	Career development	1.13	0.85-1.505
	Recognition	2.86**	2.02-4.04
	Responsibility	1.36*	1.02-1.81
	Finance	0.72**	0.56-0.92

※Strongly satisfied and satisfied coded as 1 vs. strongly dissatisfied and dissatisfied coded as 0.

*p<0.05,**p<0.01

For peer review only

For peer review only

Work stress, work motivation and their effects on job satisfaction for community health workers: A cross-sectional survey in China

Li Li*[1], Hongyan Hu[2], Hao Zhou[3], Changzhi He[1], Lihua Fan[1], Xinyan Liu[1], Zhong Zhang, Heng Li[1] ,Tao Sun[1]

[1] Department of Health Management, School of Public Health, Harbin Medical University, Harbin, China

[2] Research Service Office, The Second Affiliated Hospital, Harbin Medical University, Harbin, China

[3] Department of Emergency, Center for Disease Control and Prevention, Harbin, China

Correspondence to Li Li: lilihmu@gmail.com

Abstract

Objective: It is well documented that both work stress and work motivation are key determinants of job satisfaction. The aim of this study was to examine levels of work stress and motivation and their contribution to job satisfaction among community health workers in Heilongjiang Province, China.

Design: Cross-sectional survey.

Setting: Heilongjiang Province, China.

Participants: The participants were 930 community health workers from

For peer review only

six cities in Heilongjiang province.

Primary and secondary outcome measures: Multistage sampling procedures were used to measure socioeconomic and demographic status, work stress, work motivation, and job satisfaction. Logistic regression analysis was performed to assess key determinants of job satisfaction.

Results: There were significant differences in some subscales of work stress and work motivation by some of socioeconomic characteristics. Levels of overall stress perception and scores on all five work stress subscales were higher in dissatisfied workers relative to satisfied workers. However, levels of overall motivation perception and scores on the career development, responsibility, and recognition motivation subscales were higher in satisfied respondents relative to dissatisfied respondents. The main determinants of job satisfaction were occupation; age; title; income; the career development， and wages and benefits subscales of work stress; the recognition, responsibility and financial subscales of work motivation.

Conclusion: The findings indicated considerable room for improvement in job satisfaction among community health workers of Heilongjiang Province in China. Health care managers and policymakers should take both work stress and motivation into consideration, as two subscales of work stress and one subscale of work motivation negatively influenced job satisfaction and two subscales of work motivation positively influenced job satisfaction.

For peer review only

Keywords: work stress; work motivation; job satisfaction; community health service

ARTICLE SUMMARY

Article focus

▪How do the overall perception and subscales of work stress and motivation relate to levels of job satisfaction?

▪Which facets of work stress and motivation are affected by socioeconomic and demographic status?

▪How do work stress and motivation influence job satisfaction among community health workers?

Key message There is considerable room for improvement in job satisfaction among community health workers, and health care managers and policymakers should take both work stress and motivation into consideration.

Strengths and limitations of this study

This study is one of the first of its kind to examine the combined effects of work stress and work motivation on job satisfaction among urban community health workers in China since the implementation of new health system reform. However, the instrument used in this study is not a commonly used international scale, there may be an inherent bias in self-report measures, and the small sample may limit the generalizability of the research findings.

For peer review only

INTRODUCTION

As the foundation of the three-tier health system in China, community health service institutions played a very important role in improving access to health care service, enhancing equity and improving health. [1-2] In 2009, the Chinese central government promulgated a new set of health system reforms and called for the development of community health services. The state established basic public health service goals, which focused on providing health education, chronic disease management, and disease prevention services for urban and rural residents. From 2009 to 2012，the number of community health service institutions increased by 6,254 and the number of visits increased by 193,949 million. Therefore, community health centers and workers thereof, are very important in the process of health system reform.

Heilongjiang Province is located in Northeast China with population of about 38.1 million. There were 410 urban community health centers (CHCs) and 366 community health stations with 13,100 health workers as of December 31, 2012.[3] On average, there were 23 and 10 medical personnel in each community health center and community health station, respectively. Since the introduction of CHCs, difficulties concerning limited resources and insufficient and poorly trained staff have been present. There were 5,416 practitioners (including assistant practitioners) in community health institutions in Heilongjiang province.[3] Based on the

For peer review only - http://bmjopen.bmj.com/site/about/guidelines.xhtml

province population and human resource planning ratios, there is an approximate shortfall of 30% in the number of general practitioners (5,416 vs. 7,620) in 2012.[4] In addition, recent reforms have expanded the scope of public health services and increased workload without equivalent increases in staffing.[5,6]

In some CHCs, general practitioners, public health physicians, and nurses have been working in teams, providing medical and basic public health services to community residents, both in the centers and during home visits. With a late start, many of these practitioners were initially hospital-based specialists, and majority of public health physicians did not have a public health background. To improve skills and knowledge, continuing medical education was compulsory and no fewer than 25 credit points were required per year. Other challenges found in the CHCs were lower wages and fewer title promotion opportunities relative to general hospitals. Limited resources and a shortage of skilled health workers created very tight bottlenecks in the provision of services, which led to many community health workers experiencing work-related stress and low motivation for work, in addition to receiving low salaries and restricted opportunities for promotion.[7-8] Many studies have shown that work stress and work motivation can greatly affect job satisfaction and, in turn, the quality and delivery of health care. However, few studies have focused on work stress and motivation and their effects on job

For peer review only

satisfaction among Chinese community health workers since the implementation of the new health system reform policy.

Work stress is of great concern to managers, employees, and other stakeholders in organizations. It is a complex phenomenon and has a multitude of definitions in a variety of theoretical models.[9] According to Lazarus and Folkman's cognitive theory of stress and coping, work stress was defined as the interaction between the individual and the environment.[10] This theory suggested that when demands from the environment exceed the available resources, the result was either stress or coping, depending on the individual's appraisal of the stressors. Karasek's demand-control model assumed that psychological strain resulted from the joint effects of work demands and the degree of decision-making freedom available to workers facing the demands.[11] The effort-reward imbalance model proposed that work stress resulted from a mismatch between high commitment and effort at work and low rewards, including salary, recognition, and career promotion.[12] Nakasis and Ouzouni defined work stress as the harmful physical and emotional responses that occur when job requirements do not match workers' capabilities, resources, and needs.[13] In general, a greater imbalance between demands and individual abilities will result in greater stress.[14] Riggio classified work stress into work task stress and work role stress.[15] Cooper and Marshall's model of job stress proposed that intrinsic requirements of the job, role within the

For peer review only

organization, career development, organizational structure and climate, and relationships at work constituted the domain of work-related stress within an organization.[16] In our study, five subscales of work stress were named based on this model. Existing research has identified heavy workload, insufficient resources, work relations, lack of professional respect, and lack of promotion opportunities as possibly the most salient work stressors for community health workers.[17-19] Long-term stress may not only be harmful to the health workers themselves but may also affect community health service centers through employee dissatisfaction, burnout, poor performance, or turnover intention.[20,21-24] Therefore, it is important to reduce work stress.

Work motivation can be defined as the degree of an individual's willingness to exert and maintain an effort towards attaining organizational goals.[25] It reflected the interactions between workers and their work environments. Nahavandi and Malekzadeh believed that motivation represented a stable mind, aspiration, or interest within the individual and can translate into action.[26] Motivation theory examined the process of motivation and explained why people at work behave the way they do in terms of efforts. Building on Vroom's expectancy-valence theory of motivation, Porter and Lawler proposed a model of intrinsic and extrinsic work motivation.[27,28] This model suggested that intrinsic and extrinsic rewards were additive, and accounted for total job satisfaction.

For peer review only

Intrinsic motivation refers to doing something for the inherent satisfaction involved and is highly autonomous (i.e., self-regulated). In contrast, extrinsic motivation means doing something in order to obtain a separable outcome (i.e. tangible or verbal rewards).[29,30] Peters identified job content and work environment, extrinsic benefits, autonomy and security, and transparency as factors in work motivation for health workers using factor analysis.[31] Patrick and Wilbroad developed a tool to measure health worker motivation and revealed that organizational commitment, conscientiousness, intrinsic job satisfaction, timeliness and attendance were the major determinants of higher motivation.[32-33] Tribolet explored the relationship between intrinsic and extrinsic motivation.[34] Hoonakker found that nurses appreciated challenges and opportunities for new learning and teamwork.[35] Pool explored the significant positive association between work motivation and job satisfaction, whereas Stringer found that intrinsic motivation was positively associated, and extrinsic motivation negatively associated with job satisfaction.[36-37]

In China, previous studies have reported that poor competency and excessive workload were key work stressors among community health workers.[7,19] Shi suggested that policymakers should focus on training and educational opportunities for primary care workers and consider ways to reduce workload stress and improve salaries. [38] Hung identified professional development, training opportunities, living environment,

For peer review only

benefits, and working conditions as the most important motivating factors for primary care providers in China.[39] Ge analyzed the relationship between work stress and job satisfaction among Chinese community health workers and reported that a degree of freedom in decision making and good workplace relationships were positive predictors of job satisfaction.[40] Chen investigated relationships between work motivation, work stress and job satisfaction in cross-strait employees in Taiwan and mainland China.[41]

The present study focused on the major factors affecting work stress and motivation identified in previous research and provided an overview of community health workers' perspectives of work stress and motivation factors.[16, 42-44] The purpose of this study was to assess the predictors of job satisfaction among community health workers in one Chinese province. A cross-sectional survey was conducted to measure levels of work stress, work motivation and job satisfaction. The key predictors of job satisfaction for community health workers were assessed with special attention devoted to work stress and motivation.

METHODS

Sample

A cross-sectional survey of community health workers was conducted from March 1 to October 31, 2013 in Heilongjiang Province, China.

For peer review only

Based on the literature about community health services in China, a multi-stage, stratified sampling design was employed to ensure that study data were provincially representative.[7,40] First, six cities (Harbin, Qiqihar, Suihua, Jiamusi, Qitaihe, and Heihe) were selected based on GDP figures and three levels of the development of the community health service. Second, 15 community health centers were randomly selected from each city. On average, there were 22 medical personnel in each of the selected community health centers. Third, 60% of general practitioners, public health physicians, nurses and other health technical staff in each center were chosen randomly, with the exception of those who were absent. The research team invited all the selected staff members to participate in the study. The questionnaire included a cover page explaining the purposes and procedures of the study. The data were collected anonymously and the respondents completed the survey questionnaires privately to ensure confidentiality. Respondents were assured that participation in the survey was voluntary, and the return of questionnaires represented informed consent. The research staff stayed at the community health center and answered respondents' questions during the process of survey completion. Respondents were able to choose the best time to complete the questionnaire, such as when they were not busy or their offices were quiet. Most completed questionnaires were collected on site by the investigator on the day of the visit. If some respondents did

For peer review only

not finish that day, investigators set a date to retrieve the questionnaires. Respondents were asked to seal the completed questionnaires into individual envelopes provided by the research team. The questionnaire was relatively brief and no private personal information was collected. There were 980 questionnaires delivered to community health workers, all of which were returned. However, 50 (5.1%) were incomplete or even blank, which left 930 valid questionnaires. This study was approved by Medical Ethic Committee of Harbin Medical University.

Assessment tools

In the present study, Porter and Lawler's intrinsic and extrinsic motivation model, and Voom's expectancy-valence motivation theory were used to analyze the relationship between work motivation and job satisfaction. Lazarus and Folkman's cognitive theory of stress and coping, and Karasek's demand-control model were used to analyze the relationship between work stress and job satisfaction.[10-11]

The study instrument was part of a self-administered questionnaire composed of four sections. Section 1 focused on respondents' socioeconomic and demographic status.

Section 2 assessed work stress. Thirty items related to work stress were developed through intensive qualitative interviews with policymakers, health care managers and community health workers, a

For peer review only

review of the literature, and an initial pilot study.[16,42] Then factor analysis, which was not discussed in this paper, yielded a five-subscale structure that comprised a total of 26 items. The five-subscale solution accounted for 69.43% of the overall variance, and was found to be internally consistent (overall Cronbach's α=0.87). Based on Cooper and Marshall's model of job stress, these five subscales of work stress were named work task and role, career development, wages and benefits, workplace relationships, and organizational structure and climate stress.[16] They individually accounted for 16.05%, 25.10%, 12.00%, 9.08% and 7.20% of the overall variance, respectively, and the Cronbach's Alpha within individual subscale ranged from 0.85 to 0.90. Respondents were asked to rate their perception of work stress on each item based on a 5-point Likert scale, very less stressful (1), less stressful (2), average (3), stressful (4) and very stressful (5). The Cronbach's alpha value for this study was 0.87.

Section 3 assessed work motivation. Twenty-one items were developed based on previous research, panel discussions, and an initial pilot study.[43-45] Then 3 items were deleted and the 18 retained items were divided into four subscales by factor analysis, which was not discussed in this paper. The four-subscale solution accounted for 65.10% of the overall variance, and was found to be internally consistent (overall Cronbach's α=0.75). The subscales were renamed based on the conceptual meaning

For peer review only

of the items and comprised: career development, recognition, responsibility, and financial motivation. They individually accounted for 21.20%, 19.40%, 14.60% and 9.90% of the overall variance, and the Cronbach's Alpha within individual subscale ranged from 0.82 to 0.89. According to Porter and Lawler's intrinsic and extrinsic motivation model, we defined career development and financial motivation as extrinsic motivation, and recognition and responsibility motivation as intrinsic motivation.[28,44] Respondents were asked to rate their motivation intensity on each item based on a 5-point Likert scale, very less strong (1), less strong (2), average (3), strong (4) and very strong (5).

Section 4 assessed job satisfaction. In this study, a single-item measure was used to measure overall job satisfaction.[46] Respondents were asked to indicate their level of job satisfaction on a 4-point Likert scale, strongly dissatisfied (1), dissatisfied (2), satisfied (3) and dissatisfied (4). During the process of data analysis, strongly satisfied and satisfied were coded as 1, while strongly dissatisfied and dissatisfied were coded as 0.

Data analysis

Survey results were analyzed using SPSS 17.0. Descriptive analyses included frequencies and percentages for categorical variables and means and standard deviations (SDs) for continuous variables. Mean differences

For peer review only

were examined using t-tests and ANOVAs for relevant subgroups. We used logistic regression to measure the key predictors of job satisfaction because the dependent variable (job satisfaction) was a binary variable, which made linear regression unsuitable.

RESULTS

Socioeconomic and demographic status of respondents

Socioeconomic and demographic status of the sample were shown in Table 1. A majority of the participants were female (74.6%). General practitioners accounted for 36% of community health workers surveyed, followed by nurses (28.8%), public health physician (19.1%). In this survey, only 18.6% of them had senior professional titles and less than half (40.2%) of them had bachelor degree or higher. Only 19.6% of them had monthly incomes of more than 3,000 RMB (where $1.00US=6.23RMB in 2012). Nearly ninety percent of respondents worked more than 40 hours per week.

Work stress and motivation according to socioeconomic and demographic factors

Results of variance analysis and further multiple comparison t-tests were shown in Table 1. There were significant differences in scores for all of the five subscales of work stress according to occupation ($p < 0.01$) and gender ($p < 0.05$), with general practitioners and men showing higher

For peer review only

levels of work stress.

Scores for the wages and benefits subscale of work stress differed significantly according to educational background ($p < 0.05$) and income ($p < 0.05$). Mid-level professionals reported significantly higher levels of stress on the work task and role subscale ($p < 0.01$) and in workplace relationships ($p < 0.05$). Participants aged 35–44 and 45–54 years reported significantly higher levels of stress on the work task and role subscale ($p < 0.01$).

The male had significant higher level recognition and financial motivation ($p<0.05$). Younger workers (<25) had significantly higher level of recognition motivation ($p<0.05$) and responsibility motivation ($p<0.05$). A higher level of recognition motivation was expressed by general practitioners ($p<0.05$).

There were no significant differences in any of the four work motivation subscale scores according to educational background, professional title, or income. Men reported significantly higher levels of recognition and financial motivation ($p < 0.05$). Younger workers (<25) reported significantly higher levels of recognition ($p < 0.05$) and responsibility motivation ($p < 0.05$). General practitioners reported higher levels of recognition motivation ($p < 0.05$).

Insert Table 1 here

For peer review only

Levels of work stress, work motivation, and job satisfaction

The mean score for overall perception of work stress was 3.11, which is slightly higher than the mid-point of 3 (Table 2). Wages and benefits (3.60) subscale of work stress ranked in the highest position, followed by work task and role (3.31), career development (2.96), organizational structure and climate (2.90), and relationships (2.75) ($F=154.9$, $p<0.001$). Statistically significant differences were noted in overall perception of stress and scores on all five work stress subscales between satisfied and dissatisfied respondents; those who were dissatisfied reported higher levels of work stress ($p < 0.001$).

Career development motivation was rated the highest level, followed by financial, recognition and responsibility motivation ($F=202.6$, $p<0.001$). Levels of overall perception of work motivation and all subscales with the exception of financial motivation were significantly different between the satisfied and dissatisfied groups of respondents, and the satisfied workers had higher levels of work motivation ($p<0.01$).

In respect to motivation, career development was rated highest, followed by financial, recognition, and responsibility motivation ($F = 202.6$, $p < 0.001$). Levels of overall perception of motivation and scores on all work motivation subscales, with the exception of financial motivation, differed significantly between the satisfied and dissatisfied

For peer review only

respondents, and the satisfied workers reported higher levels of work motivation ($p < 0.01$).

Insert Table 2 here

Predictors of job satisfaction

In this study, 61.3% of respondents were satisfied with their jobs. Table 3 presented results of a logistic regression model that examined the key predictors of job satisfaction, with special attention devoted to work stress and work motivation.

Only a few demographic characteristics were predictors of job satisfaction. We found that when scores on the career development and wages and benefits subscales of work stress increased by one grade, job satisfaction decreased by 32% (odds ratio [OR] = 0.68, $p < 0.05$) and 37% (OR = 0.63, $p < 0.01$), respectively. When financial motivation increased by one grade, job satisfaction decreased by 28% (OR = 0.72, $p < 0.01$), and when recognition motivation and responsibility motivation increased by one grade, job satisfaction increased 1.86 (OR = 2.86, $p < 0.01$) and 0.36 times (OR = 1.36, $p < 0.05$), respectively. Compared with nurses, general practitioners (OR = 0.56, $p < 0.01$) and public health physicians (OR = 0.42, $p < 0.05$) reported lower job satisfaction, while other technical staff (OR = 1.89) reported higher job satisfaction. Workers

For peer review only

with no title (OR = 7.02, $p < 0.05$) were more satisfied than workers with a senior title.

Insert Table 3 here

Discussion

Job satisfaction in community health workers is important for the sustainable development of basic healthcare in China, but health policymakers and managers have neglected it for a long time.[47] This study was one of the first of its kind to examine the level of work stress and work motivation and their combined effects on job satisfaction among urban community health workers in China since the implementation of new health system reform.

Results indicated that the wages and benefits subscale of stress ranked highest, followed by the work task and role subscale. Similarly, previous research related to work stress found that low salary, heavy workload, and few promotion opportunities were the most frequently cited workplace stressors.[49,50] Several reasons may have contributed to these findings. In Heilongjiang Province, the average annual income of health service personnel in urban hospitals was 52,564 RMB ($1.00US = 6.23RMB) in 2012. In this study, 80.4% of the respondents' annual incomes were lower than 36,000 RMB. These low salaries for community

For peer review only

health workers increased their wages and benefits stress.[48] In the meanwhile, based on the province population and human resource planning ratios, there is an approximate shortfall of 30% in the number of general practitioners in 2012.[4] And the recent reforms have expanded the scope of public health services and increased workload without equivalent increases in staffing.[5,6]

Unfortunately, the present study found that scores on the career development, and wages and benefits subscales of work stress were negatively associated with job satisfaction. These findings were consistent with previous studies in which workers were likely to report low job satisfaction if they did not receive promotion and advancement opportunities or adequate salaries.[22,33,52]

With regard to work motivation, results showed the career development and financial subscales of work motivation ranked first and second respectively. Consistent with Hung and Hou's study, which found income, benefits, and professional development were the most important motivating factors among community health workers in China.[39,51]

In this study, we defined career development and financial motivation as extrinsic motivation and recognition and responsibility motivation as intrinsic motivation based on the literature.[37,53] Results reported that the recognition and responsibility subscales of work motivation were positive predictors of job satisfaction, and financial

For peer review only

motivation was a negative predictor. This was consistent with the "crowding-in" effect, which proposes that intrinsic motivation increases job satisfaction, whereas extrinsic motivation decreases job satisfaction.[54] It should be noted that in this study, the level of extrinsic motivation was higher than that of intrinsic motivation.

These findings have significant implications for managers of community health centers and policymakers in their efforts to improve workers' job satisfaction. First, policymakers should take measures to improve community health workers' salaries. In China, basic public health services are funded by the government and provided by community health workers without cost to residents. If health workers are dissatisfied with their salaries, they may prefer to work for profit-making medical services instead of nonprofit public health services. In the meanwhile, managers should implement appropriate performance salary distribution system to arouse the enthusiasms of the staff and reduce their financial stress. Second, policymakers should focus on appropriate promotion policies for community health workers. At present, it was difficult for community health workers to get title promotion, for there were limit promotion quotas for CHCs every year in Heilongjiang Province and our study found only 18.6% of respondents had senior professional title. Third, the managers should provide and support their workers to attend training or continuing education. Fourth, managers and

For peer review only

policymakers should take measures to inspire intrinsic motivation in workers. Becchetti proposed that when workers do not work for financial incentives, they may find satisfaction irrespective of their salaries, even if the financial incentive is kept to a minimum, and may therefore be satisfied with their jobs.[53] Therefore, managers and policymakers should introduce more incentives to encourage community health workers to work for responsibility or recognition.

As some subscales of work stress and work motivation can influence job satisfaction either positively or negatively, we examined levels of work stress and motivation according to demographic characteristics and found that policymakers and managers should pay more attention to three types of workers. The first group of workers included those aged between 35 and 54 years (35–44 and 45–54 age groups), who reported higher levels of stress on the work task and role subscale and lower levels of intrinsic motivation. Similar results have been reported; in Qu's study, community health workers in mid-level age groups were significantly more stressed than those in the youngest age group in one province of China.[55] This could be related to workload or difficulty and complexity of the work task, which is usually greater for 35- to 54-year-old workers, as they are the backbone of community health services. Men form the second group that requires attention. In our study, men's scores on all of the work stress subscales tended to be higher than women's, and men

For peer review only

reported higher levels of financial motivation.[56] However, another Chinese study of primary health workers found no differences in financial motivation.[51] David found that women reported more stress in the financial rewards and role ambiguity subscales.[57] The final group of workers identified as requiring attention consists of general practitioners, who experienced the highest stress according to all five work stress subscales and reported the highest career development motivation. General practitioners in community health centers face more difficult and complicated tasks and types of medical risk than other health care workers do, and they receive lower salaries and fewer promotion opportunities than their counterparts in general hospitals.

Limitations of this study

The findings in this study should be viewed in light of four key limitations. First，this study was based on a small sample of community health workers, which may limit the generalizability of the research findings. Based on the literature about community health services in China, a multistage, stratified sampling design was employed to ensure that study data were provincially representative. [7,40] A multistage, stratified sampling design was used to ensure that study data were provincially representative. Six sample cities were selected to account for the variability in regional per capita gross domestic product, and the

For peer review only

levels of healthcare development and 15 CHCs in each city were selected randomly. On average, there are 23 medical technical personnel in a community health center in Heilongjiang province and there were approximately 22 health workers in each of the community health centers in our study. In addition, the proportions of general practitioners, public health physicians, nurses, and other medical technical personnel in this study were close to the proportions found in the province as a whole.[3] Consequently, this sample was representative of Heilongjiang community health service providers, thereby enhancing the potential for generalization of the study findings. Second, the instrument for assess the work stress and work motivation was developed from earlier study and discussed with experts, while not an international commonly scale. Third, we used a cross-sectional survey, which may limit our ability to identify causal relationships between work stress and motivation and job satisfaction. Fourth, the measurements were conducted by self-administrated method and respondents' cognition can be affected by emotions at that point in time. So the common method bias and self-administrated bias might affect the results.

Conclusion

It is important for healthcare managers to improve the job satisfaction of health workers in low-resource settings. In this study, we examined levels

For peer review only

of work stress and motivation according to demographic characteristics and in respect to levels of job satisfaction; additionally, the key predictors of job satisfaction were identified using logistic regression analysis. The results indicated that community health workers rated wages and benefits highest among five subscales of work stress, and workers' extrinsic motivation was higher than their intrinsic motivation. The career development, and wages and benefits subscales of work stress and financial motivation were significant negative predictors of job satisfaction, whereas the recognition and responsibility subscales of work motivation were significant positive determinants.

Our findings suggest that there is considerable room for improvement in the job satisfaction of community health workers in Heilongjiang Province, and health care managers and policymakers should take both work stress and work motivation into consideration. First, they should pay more attention to three types of worker, as these particular groups reported higher work stress and extrinsic motivation. Second, they should take a variety of measures to reduce career development, and wage and benefits stress, as they were negative determinants of job satisfaction. Third, it is important for managers and policymakers to inspire workers' intrinsic motivation, as it can have a positive influence on job satisfaction.

Table 1 Facets of work stress and work motivation by socio-economic and demographic status for respondents

			Work stress					Work motivation			
	N	%	work task and role	career development	wages and benefits	Workplace relationships	organizational structure and climate	recognition	career development	responsibility	Finance
**Occupation**											
**General practitioner**	335	36.0	3.53	3.17	3.78	2.90	3.14	3.61	4.20	3.44	4.15
**Public health physician**	178	19.1	3.20	2.89	3.70	2.63	2.96	3.57	4.11	3.67	4.06
**Nurse**	267	28.8	3.24	2.95	3.54	2.76	2.78	3.53	4.05	3.39	4.01
**Other**	150	16.1	3.09	2.79	3.45	2.65	2.84	3.59	4.11	3.40	4.03
**F**			6.91**	4.97**	3.45**	3.05**	6.25**	0.66	2.31*	1.96	0.99
**Sex**											
**Male**	236	25.4	3.44	3.10	3.77	2.88	3.12	3.71	4.18	3.50	4.19
**Female**	694	74.6	3.27	2.93	3.56	2.72	2.85	3.56	4.12	3.43	4.03
**F**			2.50*	2.27*	2.60*	2.51*	4.09*	2.36*	1.23	1.04	2.39*
**Educational background**											
**High school or below**	110	11.8	3.18	2.90	3.36	2.81	2.72	3.57	4.13	3.42	4.05
**Junior college**	446	48.0	3.28	2.94	3.61	2.74	2.86	3.57	4.14	3.49	4.02
**College and above**	374	40.2	3.36	3.00	3.65	2.73	3.16	3.60	4.11	3.39	4.11
**F**			2.30	0.66	4.21*	0.45	4.02*	0.13	0.24	1.53	1.33
**Age in years**											
**<25**	78	8.4	3.08	2.81	3.45	2.60	2.77	3.80	4.23	3.60	3.92
**25-34**	258	27.7	3.21	2.94	3.63	2.72	2.91	3.65	4.11	3.48	4.17
**35-44**	329	35.4	3.36	2.98	3.55	2.78	2.88	3.52	4.13	3.35	4.03
**45-54**	234	25.2	3.43	3.02	3.69	2.79	2.94	3.53	4.13	3.51	4.04
**⩾55**											

For peer review only

**F**	31	3.3	3.12	2.88	3.54	2.71	2.93	3.48	3.98	3.28	3.91
			4.71**	1.01	1.36	1.12	0.83	2.89*	1.83	2.86*	2.39
**Title**											
**Senior title**	42	4.5	3.12	3.11	3.38	2.69	2.73	3.37	3.97	3.55	3.96
**Vice-senior title**	131	14.1	3.32	2.92	3.65	2.63	2.93	3.46	4.05	3.25	4.03
**Middle title**	399	42.9	3.43	3.03	3.69	2.85	2.94	3.56	4.16	3.44	4.06
**Primary title**	299	32.2	3.20	2.93	3.54	2.72	2.87	3.62	4.12	3.49	4.08
**No title**	59	6.3	3.23	2.86	3.48	2.58	2.89	3.73	4.16	3.49	4.04
**F**			3.96**	1.07	1.71	3.04*	0.59	1.73	0.98	2.13	0.16
**Monthly income （RMB）**											
**<2000**	361	38.9	3.24	2.95	3.69	2.76	2.90	3.61	4.15	3.49	4.09
**2000-2999**	386	41.5	3.32	2.96	3.61	2.75	2.88	3.59	4.13	3.40	4.06
**3000-3999**	139	14.9	3.43	2.97	3.44	2.68	2.96	3.52	4.02	3.43	3.97
**⩾4000**	44	4.7	3.39	3.03	3.21	2.93	2.78	3.44	4.27	3.53	4.28
**F**			2.11	0.99	3.14*	2.11	0.99	0.54	1.87	0.86	1.36
**Working hours (per week)**											
**<40 小时**	110	11.8	3.27	2.82	3.52	2.82	2.94	2.96	4.14	3.56	3.91
**40-47 小时**	509	54.7	3.26	2.73	3.59	2.73	2.95	2.87	4.10	3.42	4.06
**48-55 小时**	250	26.9	3.36	2.71	3.62	2.71	2.93	2.89	4.19	3.46	4.12
**⩾56 小时**	61	6.6	3.52	2.93	3.75	2.93	3.36	3.13	4.16	3.43	4.14
**F**			0.06	0.20	0.48	0.20	0.01*	0.11	0.39	0.44	0.13

*p<0.05 **p<0.01

For peer review only

Table 2 Mean scores of the overall perception and subscales of work stress and work motivation in respect to the level of job satisfaction

	Mean±SD	Level of job satisfaction		
	Total (n=930)	Satisfied (n=570, 61.3%)	Dissatisfied (n=360, 38.7%)	P
**Work stress**				
**Overall perception** ⁎	3.11±0.68	2.95±0.68	3.37±0.60	P=0.000
**work task and role¶**	3.31±0.81	3.18±0.82	3.52±0.76	P=0.000
**career development¶**	2.96±0.87	2.79±0.85	3.22±0.83	P=0.000
**Wages and benefits ¶**	3.60±0.95	3.38±0.94	3.95±0.85	P=0.000
**Workplace relationships¶**	2.75±0.79	2.61±0.79	2.96±0.74	P=0.000
**organizational structure and climate¶**	2.90±0.79	2.74±0.79	3.15±0.71	P=0.000
**Work motivation**				
**Overall perception**◎	3.80±0.55	3.86±0.55	3.70±0.55	P=0.000
**Career development**†	4.13±0.57	4.24±0.51	3.95±0.62	P=0.000
**Recognition**†	3.58±0.77	3.66±0.77	3.45±0.77	P=0.000
**Responsibility**†	3.45±0.77	3.53±0.77	3.32±3.52	P=0.000
**Financial**†	4.06±0.79	4.02±0.79	4.12±0.80	P=0.295

⁎ Mean score of overall perception of work stress was calculated for each respondent by adding the value of each item of work stress and then divided by the numbers of all item.

◎Mean score of overall perception of work motivation was calculated for each respondent by adding the value of each item of work motivation and then divided by the numbers of the item.

¶Mean score of each subscale of work stress was calculated for each respondent by adding the value of each item belongs to the subscale of work stress and then divided by the numbers of the item.

†Mean score of each subscale of work motivation was calculated for each respondent by adding the value of each item belongs to the subscale of work motivation and then divided by the numbers of the item.

For peer review only

Table 3 The logistic regression analysis for job satisfaction※

		Odds Ratio	95% CI
Occupation (Reference : nurse)	General practitioner	0.56**	0.38-0.81
	Public health physician	0.42*	0.20-0.87
	Other technical staff	1.89*	1.04-3.44
Sex (Reference :male)	Female	1.27	0.83-1.95
Educational background (Reference :High school or below)	Junior college	0.76	0.43-1.34
	College and above	0.75	0.41-1.40
Age in years (Reference : <25)	25-34	0.60	0.30-1.21
	35-44	1.10	0.51-2.42
	45-54	1.04	0.45-2.35
	⩾55	8.53**	1.86-39.01
Title (Reference : senior title)	Vice-senior title	1.86	0.476-7.29
	Middle title	2.57	0.67-9.78
	Primary title	3.84	0.96-15.39
	No title	7.02*	1.53-32.12
Monthly income in RMB (Reference : <2000)	2000-2999	0.50	0.26-0.98
	3000-3999	0.99	0.64-1.52
	⩾4000	1.30	0.86-1.97
Weekly hours worked (Reference :<40)	40-47	0.90	0.59-1.37
	48-55	1.07	0.67-1.70
	⩾56	1.20	0.62-2.33
Work stress	work task and role	0.98	0.74-1.300
	career development	0.68*	0.49-0.94
	wages and benefits	0.63**	0.50-0.79
	Workplace relationships	0.80	0.59-1.09
	Organizational structure and climate	0.97	0.71-1.33
Work motivation	Career development	1.13	0.85-1.505
	Recognition	2.86**	2.02-4.04
	Responsibility	1.36*	1.02-1.81
	Finance	0.72**	0.56-0.92

※Strongly satisfied and satisfied coded as 1 vs. strongly dissatisfied and dissatisfied coded as 0.

*p<0.05,**p<0.01

For peer review only

Acknowledgements We are thankful to all the community health workers who participated in the study. We are also grateful to Yin Li, Xingsan Li, Zhuang Wang and Hongjuan Wei, who have worked closely with the team to ensure the field survey is successfully implemented.

Completing interests None.

Contributors

LiLi was responsible for the study design, data analysis and the drafting and revising of the manuscript. HongyanHu and ChangzhiHe, who contributed equally as the first author to this article, were responsible for study design, data collection and data analysis. HaoZzhou and ZhongZ hang provided statistical expertise. XinyanLiu, TaoSun and HengLi performed data collection and technical support. LihuaFan provided administrative support. All authors read and approved the final manuscript.

Funding This study was funded by the National Science Foundation of China (NSFC), Contract No.71203050/G0308, Contract No.71073034 and was supported by Young Seed Foundation of Public Health College of Harbin Medical University. The opinions expressed herein are the authors' and do not necessarily reflect the views of NSFC and the survey was conducted independently by researchers from Harbin Medical University.

For peer review only

References

1. Starfield B, Shi LY, Macinko J. Contribution of primary care to health systems and health. Milbank Q 2005;83: 457–502.
2. Hung LM, Rane S, Tsai J, et al. Advancing primary care to promote equitable health: implications for China. Int J Equity Healt 2012;11:2.
3. Heilongjiang Provincial Bureau. Heilongjiang Health Statistical Yearbook 2012. Harbin, China: Heilongjiang Provincial Press 2013.
4. State Commission Office for Public Sector Reform. (2006). Notice on Issuing the Instruction Opinions on Organization Structure and Staffing of Urban Community Health Service. Beijing, China. http://www.gov.cn/zwgk/2006-09/04/content_377067.htm (accessed on 4 September 2006).
5. MOH (Ministry of Health of China) Chinese Health statistics in 2010. Peking Union Medical College Press 2010.
6. MOH (Ministry of Health of China) (2012) Chinese Health statistics in 2010. Peking Union Medical College Press.
7. Zhou W, Dong YM, Lin XZ, et al. Community health service capacity in China: a survey in three municipalities. J Eval Clin Pract 2013;19(1):167–17.
8. Li L, Zhou H, Yao GF, et al. Analysis on working motivation of community health workers in Harbin, China. Medicine and Society (in Chinese) 2013;7:40-43.

For peer review only

9. Clegg A. Occupational stress in nursing: a review of the literature. J Nurs Manage 2001; 9:101–106.

10. Lazarus RS. & Folkman S. Stress, Appraisal and Coping. Springer, New York, NY.1984.

11. Karasek RA. Job demands, job decision latitude, and mental strain: implications for job re-design. Administrative Science Quarterly 1979; 24(2): 285–308.

12. Siegrist J. Adverse health effects of high-effort/low-reward conditions. J Occup Health Psychol 1996; 1:27–41.

13. Nakasis K., Ouzouni C. Factors influencing stress and job satisfaction of nurses working in psychiatric units: a research review. Heal Sci J 2008;2 (4): 183–195.

14. Jamal M. Burnout among Canadian and Chinese employees: a cross-cultural study. European Management Review 2005;2(3):224–230.

15. Riggio R. Introduction to industrial organizational psychology. New Jersey: Pearson Education, Inc. 2003.

16. Cooper CL, Marshall J. Occupational sources of stress: a review of the literature relating to coronary heart disease and mental ill health. J Occup Psychol 1976; 49:11-28.

17. Kim HJ. Work Stress and Job Satisfaction of Community Mental Health Nurses in South Korea: A Qualitative Content Analysis. J

For peer review only

Korean Acad Psychiatr Ment Health Nurs 2013;22(4):295-306.

18. Johnson SJ, O'Connor EM, Jacobs S, et al. The relationships among work stress, strain and self-reported errors in UK community pharmacy. Research in Social and Administrative Pharmacy. 2014;1.
19. Qu NQ, Zhou H, Fan LH, et al. Analysis on work stress and occupational burnout among community health service staff in Harbin. Medicine and Society 2013;26(9):87-89.
20. Chen XJ, Tan XR, Li LP. Health Problem and Occupational Stress among Chinese Doctors. Chinese Medicine 2013;4: 1-6.
21. Nabirye RC, Brown KC, Pryor ER et al. Occupational stress, job satisfaction and job performance among hospital nurses in Kampala, Uganda. J Nurs Manage 2011; 19:760–768.
22. Voltmer E, Rosta J, Johannes Siegrist, et al. Job stress and job satisfaction of physicians in private practice: comparison of German and Norwegian physicians. Int Arch Occ Env Hea 2012;85,(7):819-828.
23. Saijo Y, Chiba S, Yoshioka E, et al. Job stress and burnout among urban and rural hospital physicians in Japan. Aust J Rural Health 2013;21(4): 225–231.
24. Sun Y, Luo ZN, Fang PQ. Factors Influencing the Turnover Intention of Chinese Community Health Service Workers Based on the Investigation Results of Five Provinces. J Commun Health

For peer review only

2013;38(6):1058-1066.

25. Franco LM, Bennett S, Kanfer R, et al. Determinants and consequences of health worker motivation in hospitals in Jordan and Georgia. Soc Sci Med 2004;58:343-355

26. Kanfer R. Measuring health worker motivation in developing countries. Partnerships for Health Reform Project, Major Applied Research Working Paper 1999.

27. Vroom, V. H. Work and motivation. New York: Wiley.1964.

28. Porter LW, Lawler EE. Managerial attitudes and performance. Homewood, IL: Irwin-Dorsey 1968.

29. Ryan RM, Deci EL. Self-determination theory and the facilitation of intrinsic motivation, social development, and wellbeing. Am Psychol 2000; 55(1): 68–77.

30. Ryan RM, Deci EL. Self-regulation and the problem of human autonomy: Does psychology need choice, self-determination, and will? J Pers 2006; 74(6):1558–1585.

31. Peters DH, Chakraborty S, Mahapatra P, et al. Job satisfaction and motivation of health workers in public and private sectors: cross-sectional analysis from two Indian states. Hum Resour Health. 2010;8:27.

32. Patrick MM, Duane B, Lucy G, et al. Developing a tool to measure health worker motivation in district hospitals in Kenya. Hum Resour

For peer review only

Health 2009; 7:40.

33. Wilbroad M, Helen A, Virginia B, et al. Measuring health workers' motivation in rural health facilities: baseline results from three study districts in Zambia. Hum Resour Health 2013;11:8.

34. Tribolet WC. The relationship between intrinsic and extrinsic motivation and organizational commitment: a study in a European environment [D]. Nova Southeastern Univ 2004.

35. Hoonakker PL, Carayon P, McGuire K, et al. Motivation and job satisfaction of Tele-ICU nurses. J Crit Care 2013;28:315.e13 -315.e21.

36. Pool SW. The relationship of job satisfaction with substitutes of leadership, leadership behavior, and work motivation. J Psychol 1997;131(3):271-83.

37. Stringer C, Jeni D, Theivananthampillai. Motivation, pay satisfaction, and job satisfaction of front-line employees. Qual Res Acco Man 2011; 8(2):161-179.

38. Shi L, Hung LM, Song K, et al. Chinese primary care physicians and work attitudes. Int J Health Serv 2013;43(1):167–181.

39. Hung LM, Shi L, Wang H, et al. Chinese primary care providers and motivating factors on performance. Fam Prac 2013; 30:576–586.

40. Ge C, Fu J, Chang Y, et al. Factors associated with job satisfaction among Chinese community health workers: a cross-sectional study.

For peer review only

BMC Public Health, 2011;24(11):884.

41. Chen TL, Huang MY, Su TH. Work motivation, work stress, and job satisfaction in between Taiwan and China-An empirical study. World Acad Sci, Eng Tech 2012; 68:1446-1450.

42. Ajayi MP., Abimbola OH. Job satisfaction, organizational stress and employee performance: a study of NAPIMS. Ife Psychol 2013;21:75-82.

43. Wayne SJ, Liden RC. Effects of impression management on performance ratings: a longitudinal study. Acad Manage J 1995;38:232-260.

44. Gagne M, Deci EL. Self-determination theory and work motivation. J Organ Behav 2005;26:331-362.

45. Dieleman M, Cuong PV, Anh, LV, et al. Identifying factors for job motivation of rural health workers in North Vietnam. Hum Resour Health 2003;1:10.

46. Wanous JP, Reichers AE, Hudy MJ. Overall job satisfaction: how good are single-item measures? J Appl Psychol 1997;82:247–52.

47. Chen J, Dong JQ, Ding J, Du XP. Analysis on satisfaction of professionals in community health service institutions held by different levels of hospital. Chin Gen Pract 2008;4(11): 631-634. (In Chinese)

48. National Bureau of Statistics. China Statistic Yearbook 2013.

For peer review only

Beijing ,China: China Sta Press. 2013.

49. Ding H, Sun X, Chang WW, et al. A comparison of job satisfaction of community health workers before and after local comprehensive medical care reform: a typical field investigation in central China. Plos One 2013;8(9):1-5.

50. Han JF, Li XH. An analysis on the job stressors of community health nurses.Chinese Nursing Management. 2007;7(5):45-48. (in Chinese)

51. Hou ZH, Meng QY, Yuan BB et al. Motivation preferences of general practices in rural China. Chinese Journal of Health Policy, 2010;13(10):18-22.

52. Lephalala RP. Factors influencing nurses job satisfaction in selected private hospitals in England. Curationis 2008;31(3): 60–69.

53. Becchetti L, Castriota S, Tortia EC. Productivity, wages and intrinsic motivations. Sma Bus Eco 2012;41:379–399.

54. Frey BS. On the relationship between intrinsic and extrinsic work motivation. Int J Ind Organ 1997;15(4),427-439.

55. Qu JW. Investigation and research on job stress and degree of loyalty in community health workers in Liaoning Province (M). Shenyang: Chinese Medical University 2013.

56. Malik AA, Yamamoto SS, Souares A, et al. Motivational determinants among physicians in Lahore Pakistan. BMC Health Serv Res 2010;10:201.

For peer review only

57. David PH, Srinika DJ. Gender differences in work stress among clinical social workers. J Soc Serv Res 2008;10:18.

For peer review only

STROBE Statement—Checklist of items that should be included in reports of ***cross-sectional studies***

	Item No	Recommendation	Reported on page #
**Title and abstract**	1	(*a*) Indicate the study's design with a commonly used term in the title or the abstract	2，7
		(*b*) Provide in the abstract an informative and balanced summary of what was done and what was found	2
**Introduction**			
Background/rationale	2	Explain the scientific background and rationale for the investigation being reported	4，,5
Objectives	3	State specific objectives, including any prespecified hypotheses	9
**Methods**			
Study design	4	Present key elements of study design early in the paper	9
Setting	5	Describe the setting, locations, and relevant dates, including periods of recruitment, exposure, follow-up, and data collection	10
Participants	6	(*a*) Give the eligibility criteria, and the sources and methods of selection of participants	10，11
Variables	7	Clearly define all outcomes, exposures, predictors, potential confounders, and effect modifiers. Give diagnostic criteria, if applicable	11-13
Data sources/ measurement	8*	For each variable of interest, give sources of data and details of methods of assessment (measurement). Describe comparability of assessment methods if there is more than one group	
Bias	9	Describe any efforts to address potential sources of bias	10
Study size	10	Explain how the study size was arrived at	10
Quantitative variables	11	Explain how quantitative variables were handled in the analyses. If applicable, describe which groupings were chosen and why	10，11
Statistical methods	12	(*a*) Describe all statistical methods, including those used to control for confounding	13，14
		(*b*) Describe any methods used to examine subgroups and interactions	
		(*c*) Explain how missing data were addressed	
		(*d*) If applicable, describe analytical methods taking account of sampling strategy	
		(*e*) Describe any sensitivity analyses	
**Results**			
Participants	13*	(a) Report numbers of individuals at each stage of study—eg numbers potentially eligible, examined for eligibility, confirmed eligible, included in the study, completing follow-up, and analysed	
		(b) Give reasons for non-participation at each stage	
		(c) Consider use of a flow diagram	
Descriptive data	14*	(a) Give characteristics of study participants (eg demographic, clinical, social) and information on exposures and potential confounders	
		(b) Indicate number of participants with missing data for each variable of interest	
Outcome data	15*	Report numbers of outcome events or summary measures	

For peer review only - http://bmjopen.bmj.com/site/about/guidelines.xhtml

Main results	16	(*a*) Give unadjusted estimates and, if applicable, confounder-adjusted estimates and their precision (eg, 95% confidence interval). Make clear which confounders were adjusted for and why they were included	
		(*b*) Report category boundaries when continuous variables were categorized	
		(*c*) If relevant, consider translating estimates of relative risk into absolute risk for a meaningful time period	
Other analyses	17	Report other analyses done—eg analyses of subgroups and interactions, and sensitivity analyses	13
**Discussion**			
Key results	18	Summarise key results with reference to study objectives	14-17
Limitations	19	Discuss limitations of the study, taking into account sources of potential bias or imprecision. Discuss both direction and magnitude of any potential bias	3，22
Interpretation	20	Give a cautious overall interpretation of results considering objectives, limitations, multiplicity of analyses, results from similar studies, and other relevant evidence	19-21
Generalisability	21	Discuss the generalisability (external validity) of the study results	17
**Other information**			
Funding	22	Give the source of funding and the role of the funders for the present study and, if applicable, for the original study on which the present article is based	29

*Give information separately for exposed and unexposed groups.

Note: An Explanation and Elaboration article discusses each checklist item and gives methodological background and published examples of transparent reporting. The STROBE checklist is best used in conjunction with this article (freely available on the Web sites of PLoS Medicine at http://www.plosmedicine.org/, Annals of Internal Medicine at http://www.annals.org/, and Epidemiology at http://www.epidem.com/). Information on the STROBE Initiative is available at www.strobe-statement.org.

For peer review only

For peer review only - http://bmjopen.bmj.com/site/about/guidelines.xhtml
